# The Oncogene PDRG1 Is an Interaction Target of Methionine Adenosyltransferases

**DOI:** 10.1371/journal.pone.0161672

**Published:** 2016-08-22

**Authors:** Claudia Pérez, Francisco J. Pérez-Zúñiga, Francisco Garrido, Edel Reytor, Francisco Portillo, María A. Pajares

**Affiliations:** 1 Instituto de Investigaciones Biomédicas Alberto Sols (CSIC-UAM), Arturo Duperier 4, 28029 Madrid, Spain; 2 Instituto de Investigación Sanitaria La Paz (IdiPAZ), Paseo de la Castellana 261, 28046 Madrid, Spain; 3 Departamento de Bioquímica, Facultad de Medicina, Universidad Autónoma de Madrid, Arzobispo Morcillo 4, 28029 Madrid, Spain; Indian Institute of Science, INDIA

## Abstract

Methionine adenosyltransferases MAT I and MAT III (encoded by *Mat1a*) catalyze S-adenosylmethionine synthesis in normal liver. Major hepatic diseases concur with reduced levels of this essential methyl donor, which are primarily due to an expression switch from *Mat1a* towards *Mat2a*. Additional changes in the association state and even in subcellular localization of these isoenzymes are also detected. All these alterations result in a reduced content of the moderate (MAT I) and high V_max_ (MAT III) isoenzymes, whereas the low V_max_ (MAT II) isoenzyme increases and nuclear accumulation of MAT I is observed. These changes derive in a reduced availability of cytoplasmic S-adenosylmethionine, together with an effort to meet its needs in the nucleus of damaged cells, rendering enhanced levels of certain epigenetic modifications. In this context, the putative role of protein-protein interactions in the control of S-adenosylmethionine synthesis has been scarcely studied. Using yeast two hybrid and a rat liver library we identified PDRG1 as an interaction target for MATα1 (catalytic subunit of MAT I and MAT III), further confirmation being obtained by immunoprecipitation and pull-down assays. Nuclear MATα interacts physically and functionally with the PDRG1 oncogene, resulting in reduced DNA methylation levels. Increased *Pdrg1* expression is detected in acute liver injury and hepatoma cells, together with decreased *Mat1a* expression and nuclear accumulation of MATα1. Silencing of *Pdrg1* expression in hepatoma cells alters their steady-state expression profile on microarrays, downregulating genes associated with tumor progression according to GO pathway analysis. Altogether, the results unveil the role of PDRG1 in the control of the nuclear methylation status through methionine adenosyltransferase binding and its putative collaboration in the progression of hepatic diseases.

## Introduction

Transmethylations encompass a large variety of reactions in which a methyl group is incorporated into a diversity of substrates, including DNA, proteins and small molecules [1, 2]. These processes require methyl donors, a role performed mainly by S-adenosylmethionine (AdoMet) in mammals [3, 4]. Synthesis of this metabolite is carried out by addition of the adenosine moiety of ATP to the methionine sulfur atom in a particular two-step reaction (requiring Mg^2+^ and K^+^ ions) catalyzed by methionine adenosyltransferases (MATs)[3]. Three MAT genes exist in mammals (*Mat1a*, *Mat2a* and *Mat2b*), which encode for two catalytic subunits (MATα1 and MATα2) and a regulatory β-subunit (MATβ)[3, 5]. *Mat1a* achieves its highest expression levels in normal liver, although small levels can be detected in almost every tissue [6]. In contrast, *Mat2a* is preferentially expressed in extrahepatic tissues and in fetal liver, a pattern also followed by *Mat2b* [3, 4, 7, 8]. Rat MATα1 and MATα2 are 85% identical at the amino acid level, reflecting the high conservation detected among α-subunits in the MAT family [9]. MATβ is an unrelated protein classified into the PFAM 04321 family of oxidoreductases [3], which regulates the activity of MATα2 oligomers by enhancing their affinity for methionine (reviewed in [3, 4]). These subunits associate to constitute three isoenzymes: the homo-oligomers MAT I and MAT III, a tetramer and a dimer of MATα1 subunits, respectively [3]; and the hetero-trimer MAT II composed by a MATα2 dimer and one MATβ subunit [10]. Structural studies have demonstrated that the minimum active assembly is a dimer of α-subunits, with both monomers contributing residues to form two active sites at their interface [4, 11–13].

Most of the hepatic diseases studied to date, including cirrhosis, hepatocellular carcinoma or acute liver injury, concur with a reduction in AdoMet concentrations due to a decrease in *Mat1a* expression and the concomitant increase in that of *Mat2a* and *Mat2b* [3, 14, 15]. Effects at the cytosolic protein level follow the same trend with increases in MATα2 and MATβ and a reduction in MATα1, which also changes its preferred association state towards MAT III [16, 17]. Data regarding the MAT nuclear pool are limited, due to their recent identification in this compartment [6, 18]. Nevertheless, a distinct regulation between cytoplasmic and nuclear levels of MATα1 is detected in rat models of acute liver injury, which show nuclear accumulation of the protein together with its cytoplasmic reduction [17]. Nuclear MAT activity rises together with MAT I content as compared to normal liver, and correlates with increased levels of histone 3 K27 trimethylation (me3K27H3), an epigenetic methylation involved in gene repression [17, 19]. Additional effects derive from the fact that most of these diseases also present with oxidative stress, and hence with enhanced production of free radicals, nitric oxide and/or changes in the GSH/GSSG ratio. These outcomes result in post-translational modifications reducing MAT I/III activity and/or alterations in the association state [20–23]. MATα2 is protected against these post-translational modifications by the lack of equivalent residues in its sequence [24, 25], but its oligomerization with MATβ is favored by the enhancement of NADP^+^ levels, hence aiding to reduce AdoMet production [10, 25].

In this context, data about the role of protein-protein interactions in MAT regulation are limited to a few studies concentrated on MATα2 and MATβ, especially in cancer cells [18, 26–28]. Altogether these results show an evident lack of information regarding putative MATα1 interactions, and hence, we have addressed this aspect searching for liver proteins able to interact with MATα1 using yeast two-hybrid screening. This approach rendered the identification of the PDRG1 oncogene as a novel interaction partner for MATα1 in the nucleus of hepatic cells.

## Materials and Methods

### Plasmids and mutagenesis

The ORF of rat *Mat1a* was obtained by NdeI/BamHI digestion of pSSRL-Blue T2 [29], and cloned into pGBKT7 (Clontech, Mountain View, CA, USA) to get pGBKT7-MAT1A. Cloning into the NcoI/BamHI sites of pACT2 (Clontech) required three steps: i) amplification of the *Mat1a* ORF from pSSRL-Blue T2 using the primers: 5’-AACATACCATGGAGATGAATGGACCTGTGGATG-3’(sense; NcoI site underlined) and 5’-AGGGAACAAAAGCTGGAGC-3’(reverse); ii) NcoI/BamHI digestion of the amplified fragment that renders the ORF in two fragments; and iii), sequential cloning of the two fragments into pACT2 to obtain pACT2-MAT1A.

One of the pACT2 plasmids containing the full ORF of rat *Pdrg1* (402 bp) was used for amplification with the primers 5’-CGGAATTCCTCTGTGGCACCATGGTGT-3’(sense; EcoRI site underlined) and 5’-CGGGATCCTCATCCTTTCAAGATGACCTGG-3’(reverse; BamHI site underlined). The amplification conditions included: i) 2 min denaturation at 95°C; ii) 30 cycles including 30s denaturation at 95°C, 1 min annealing at 56°C and 1 min extension at 72°C; and iii) 10 min extension at 72°C. The amplified fragment was cloned into pBluescript SK+, rendering pBS-PDRG1. EcoRI/NotI digestion of pBS-PDRG1 allowed subcloning of the insert into pHA(del), lacking C876 of pCMV-HA [17], and pGEX-5X-1 (GE Healthcare, Uppsala, Sweden) to produce pHA-PDRG1 and pGEX-PDRG1, respectively. The HA-tagged PDRG1 contained 22 additional residues at the N-terminal (MYPYDVPDYALMAMEAEFLCGT; HA-tag underlined), whereas the GST-PDRG1 construct included a 9 amino acid linker (GIPEFLCGT) between the fused proteins. Cloning into pEGFP-N1 (BD Biosciences, San Jose, CA, USA) required amplification of the *Pdrg1* ORF from pACT2-PDRG1, as described above, using the same sense primer and a new antisense primer to eliminate the stop codon (5’-CGGGATCCCGTCCTTTCAAGATGACCTGGAG-3’). The amplified fragment was EcoRI/BamHI digested and cloned into pEGFP-N1 (Clontech), rendering pPDRG1-EGFP. The corresponding fused protein contains a 7 amino acid linker (RDPPVAT) between PDRG1 and EGFP. A NdeI restriction site was included on pBS-PDRG1 using the QuikChange method (Stratagene, La Jolla, CA, USA), the sense primer 5’-CTGTGGCACCATATGGTGTCCCCCGAG-3’ and its complementary. NdeI/BamHI digestion of the modified pBS-PDRG1 allowed subcloning of the insert into pT7.7 (Dr. Stan Tabor, Harvard Medical School, Boston, MA, USA) to obtain pT7.7-PDRG1. The ORF of rat *Pdrg1* was extracted from pT7.7-PDRG1 by NdeI/PstI digestion and subcloned into pTYB12 (New England Biolabs, Beverly, MA, USA) to obtain pTYB12-PDRG1.

PDRG1 deletion mutants were generated at the N-terminal (ΔN), C-terminal (ΔC) and both ends (ΔNC). For this purpose, amplification from pBS-PDRG1 using the primers 5’-GGAATTC**ATG**GACAAGCGGCAGATTGTAGACC-3’(sense; EcoRI site underlined) and 5’-ATAAGAATCGGCCGCTCTAGAACTAGTGGATCC-3’(reverse; NotI site underlined) was carried out, to obtain an ORF lacking bases 4–75 of the N-terminal. The amplified fragment was digested and cloned into the EcoRI/NotI sites of pGEX-5X-1, to obtain pGEX-ΔN-PDRG1. Plasmids pGEX-PDRG1 and pGEX-ΔN-PDRG1 were used to include a new stop codon at position 321–323 by the QuikChange method, the primer 5’-CTCCTAGAAGCCCAATGAAAACCGGAGCTAAAGG-3’and its complementary, rendering pGEX-ΔC-PDRG1 and pGEX-ΔNC-PDRG1, respectively.

Additional plasmids used in this work include: pFLAG-MAT and pSSRL-T7N containing the rat *Mat1a* ORF [6, 30]; pT7.7-MAT2A including the human *MAT2A* ORF [10]; pT7.7-MAT2B containing the human *MAT2B* ORF [10]; and pTYB12-MAT2B that was generated by NdeI/EcoRI digestion of pT7.7-MAT2B and cloning of the insert into pTYB12. Human MATα2 and MATβ subunits are 98% and 95% identical to their rat homologues. The presence of the correct sequences in all the plasmids described above was verified by automatic sequencing at the Genomic Service of the Instituto de Investigaciones Biomédicas “Alberto Sols” (IIBM, CSIC-UAM).

### Yeast two hybrid

A rat liver Matchmaker cDNA library (RL4004AH; Clontech) and the AH109 yeast strain were used for screening in search for MATα1 interactions. Yeast transformations were carried out using EasyComp solutions (Invitrogen, Carlsbad, CA, USA) and the resulting transformants grown in low (-Leu/-Trp; -LW) and high stringency (–Ade/-His/-Leu/-Trp; -AHLW) SC media for selection. Screening of 6.5 x10^5^ clones was carried out and 41 putative interactions detected, from which only 23 were confirmed in–AHLW SC medium. DNAs of the positive clones were isolated and used to transform *E*. *coli* DH5α competent cells. Plasmids were purified using Qiagen plasmid purification kits (Qiagen, Hilden, Germany) and sequenced. Eight biologically relevant preys were found, the rest corresponding to MATα1-MATα1 interactions; this large background was expected for a homo-oligomeric protein and guarantees native MATα1 folding of the fusion proteins. Verification of positive interactions was performed by cotransformation of plasmids harboring *Mat1a* ORF and putative preys, followed by growth on–AHLW SC media.

### Cell culture, transfections and confocal microscopy

Commercial CHO (chinese hamster ovary), COS-7 (monkey kidney), H35 (rat hepatoma), N2a (mouse neuroblastoma) and HEK-293T (human kidney) cell lines were obtained from the ATCC and IIBM collections and grown in DMEM (Gibco, Grand Island, NY, USA) supplemented with 10% (v/v) fetal bovine serum and 2 mM glutamine. Transient transfections with pHA-PDRG1, pPDRG1-EGFP or pFLAG-MAT were carried out for 48 hours using lipofectamine (Invitrogen), as previously described [6]. Experiments requiring cotransfection included pHA-PDRG1 or pPDRG1-EGFP and pFLAG-MAT.

Direct fluorescence observation (40000–100000 cells) and immunofluorescence (10000–40000 cells) were carried out with transiently transfected cells grown on glass coverslips as previously described [6]. Nuclei were stained using 5 μg/ml Hoechst 33342 dye (Molecular Probes, Eugene, OR, USA) for 1 hour before direct observation or fixation. Minor modifications concerned the use of 5 min fixation and permeabilization steps. The antibodies and dilution used for immunofluorescence are listed in Table 1. Glass coverslips were mounted using Prolong (Molecular Probes). Cell imaging (0.3–0.4 μm sections) was performed on a Leica TCS SPII Spectral microscope using a 63x /1.3 NA objective. Images were analyzed using the Leica Confocal Software (LCS Lite, Zurich, Switzerland).

**Table 1:** Antibodies used in this work.

Primary antibody (Source)	Dilution (v/v)^1^	Secondary antibody	Dilution (v/v)	Application^2^
Rabbit anti-MATα1 (Mingorance et al. [29])	1:10000	Goat anti-rabbit IgG (BioRad; 170–6515)	1:10000	WB
Rabbit anti-MATα1 (Mingorance et al. [29])	1:1000	Goat anti-rabbit Alexa Fluor 546 (Molecular Probes; A11035)	1:400	IF
Rabbit anti-MATβ (Abcam; ab109484)	1:2000	Goat anti-rabbit IgG (BioRad; 170–6515)	1:10000	WB
Mouse anti-FLAG (Sigma; F3165)	5 μg/ml	Anti-mouse IgG (GE Healthcare; NA931)	1:20000	WB
Mouse anti-FLAG (Sigma; F3165)	5 μg/ml	Goat anti-mouse Alexa Fluor 488 (Molecular Probes; A11029)	1:400	IF
Mouse anti-TBP (Santa Cruz; sc-56796)	1:1000	Anti-mouse IgG (GE Healthcare; NA931)	1:20000	WB
mouse anti-tubulin (Sigma; T9026)	1:2500	Anti-mouse IgG (GE Healthcare; NA931)	1:20000	WB
Mouse anti-HA (Covance; MMS-101R)	1:1000	Anti-mouse IgG (GE Healthcare; NA931)	1:20000	WB
Mouse anti-HA (Covance; MMS-101R)	1:1000	Goat anti-mouse Alexa Fluor 488 (Molecular Probes; A11029)	1:400	IF
Mouse anti-GST (Cell Signaling; #2624)	1:10000	Anti-mouse IgG (GE Healthcare; NA931)	1:20000	WB
Mouse anti-SC35 (BD PharMingen; 556363)	1:1000	Goat anti-mouse Alexa Fluor 546 (Molecular Probes; A11030)	1:400	IF
Chicken anti-MATα2 (Abcam; ab26174)	1:10000	Goat anti-chicken IgY (Abcam; ab6877)	1:2000	WB
Rat anti-HA (Roche; 11-867-423-001)	100 ng/ml	Goat anti-rat Alexa Fluor 488 (Molecular Probes; A11006)	1:500	IF
Rabbit anti-PDRG1 (Abcam; ab121219)	1:1000	Goat anti-rabbit IgG (BioRad; 170–6515)	1:1000	WB^3^

^1^Dilution expressed as (v/v), except where otherwise indicated

^2^WB, western blotting; IF, immunofluorescence.

^3^Exposed on ultrasensitive film.

### Subcellular fractionation and immunoprecipitation

Total lysates were prepared from 2 x 10^6^ cells in 200 μl of 50 mM Tris/HCl pH 7.5, 150 mM NaCl, 1 mM EDTA, 1% (v/v) NP-40, 1 mM DTT containing protease inhibitors (1 mM PMSF, 1 mM benzamidine, 2 μg/ml aprotinin, 1 μg/ml pepstatin A, 0.5 μg/ml leupeptin, 2.5 μg/ml antipain), after 10 min incubation on ice, by a 6-fold passage through a needle. Input samples (50 μl) were taken at this step and the remaining sample centrifuged for 15 min at 10000 xg at 4°C. When subcellular fractionation was required either from liver or cells, nuclear and cytoplasmic fractions were prepared as previously described [6]. For anti-HA immunoprecipitation, total lysates (150 μl), nuclear (100 μl) or cytosolic fractions (100 μl) were precleared using anti-mouse IgG (2 μg) for 2 hours at 4°C and centrifuged for 15 min at 10000 xg. The supernantants were incubated overnight with anti-HA (2 μg) coupled to protein A Sepharose CL-4B (GE Healthcare) at 4°C. Anti-FLAG immunoprecipitation was carried out overnight at 4°C by incubation of the subcellular fractions with anti-FLAG M2 Affinity Gel (50 μl; Sigma, Madrid, Spain; A2220). The beads were washed 3 times and boiled in Laemmli sample buffer (40 μl) containing 100 mM DTT for 10 min. Samples were centrifuged for 1 min at 10000 xg, the supernatants loaded on SDS-PAGE gels and proteins were transferred to nitrocellulose membranes for western blotting.

### Western blotting

Immunoblotting was carried out as described previously [29] using the antibodies and conditions listed in Table 1. For FLAG-MATα1 detection, after anti-HA immunoprecipitation, mouse TrueBlot ULTRA (1:1000 v/v; eBioscience, San Diego, CA, USA; 18–8817) was used. Protein bands were visualized using Western Lightning^™^ chemiluminescence reagent (Perkin Elmer, Waltham, MA, USA).

### Analytical gel filtration chromatography

HEK 293-T cells (4 x 10^6^) transiently transfected with the plasmids of interest were used to obtain the nuclear fractions as previously described [6]. Samples (100 μl) of the nuclear fractions were injected on a Superose 12 10/300 GL column (GE Healthcare), and elution performed as previously described [23]. Dot-Blot analysis of the fractions (100 μl) was performed using the same conditions than for western blot. The protein standards (GE Healthcare and Sigma) used were: Blue dextran (2000 kDa); ferritin (440 kDa); β-amylase (200 kDa); aldolase (150 kDa); alcohol dehydrogenase (150 kDa); conalbumin (75 kDa); ovalbumin (43 kDa); carbonic anhydrase (29 kDa); lysozyme (14.4 kDa); and ATP (551 Da).

### Production of recombinant proteins

The recombinant proteins used in this study were overexpressed in *E*. *coli* BL21(DE3) Codon Plus cells using the specific conditions described in Table 2. Refolding and purification of recombinant MATα1 and MATα2 from inclusion bodies was carried out as previously described [10, 30]. Purification of MATβ and PDRG1 was performed using soluble fractions and chitin beads (New England Biolabs) as previously described for betaine homocysteine methyltransferase [31], but using 50 mM β-mercaptoethanol for 60 hours at room temperature (23°C) for tag excision. Purification of GST-PDRG1 was carried out using Glutathione-Sepharose 4B (GE Healthcare) following manufacturer's instructions and the protein was eluted with 20 mM GSH. This tagged-PDRG1 was preferred when detection by western blotting or dot-blot was required. MAT II (α2_2_β) was produced by incubation of equimolar concentrations of both recombinant subunits for 1 hour at 4°C, as described by González et al. [10]. Similarly, oligomers containing MATα1 or MATα2 and GST-PDRG1 were obtained by incubation of equimolar concentrations of the purified recombinant proteins for 1 hour at 4°C, followed by gel filtration chromatography on Biogel A columns (1.5 x 90 cm; Bio-Rad, Hercules, CA, USA), equilibrated and run with 50 mM Tris/HCl pH = 8, 10 mM MgSO_4_, 50 mM KCl at 10 ml/h and 4°C. A_280_ was detected during elution and MAT activity was measured in the collected fractions (3 ml). Samples (1 ml) of each fraction were precipitated with TCA and loaded onto SDS-PAGE gels, where the presence of both proteins was detected after Coomasie blue staining and/or western blotting of the pooled activity peaks. The protein standards (GE Healthcare and Sigma) used were: Blue dextran (2000 kDa); ferritin (440 kDa); aldolase (150 kDa); conalbumin (75 kDa); ovalbumin (43 kDa); and ATP (551 Da).

**Table 2:** Conditions for the expression of recombinant proteins.

	Induction^1^		
plasmid	DO_600_	Time (h)^2^	Temperature (°C)
pGEX-5X-1	0.3–0.4	20	20
pGEX-PDRG1	0.3–0.4	6	20
pGEX-ΔN-PDRG1	0.3–0.4	9	20
pGEX-ΔC-PDRG1	0.3–0.4	9	20
pGEX-ΔNC-PDRG1	0.3–0.4	6	20
pSSRL-T7N	0.3–0.4	3	37
pT7.7-MAT2A	0.3–0.4	20	27
pTYB12-MAT2B	0.5–0.6	4	20
pTYB12-PDRG1	0.5–0.6	20	20

^1^ Induction with IPTG (0.5 mM final concentration) when the desired D.O. at 600 nm was achieved

^2^ Cultures were continued for the time length and temperature specified.

### Pull-down

Bacterial pellets overexpressing the protein of interest were lysed by sonication (5 cycles of 30s on/off) in 1:5 (w/v) PBS buffer containing 300 mM NaCl, 10 mM DTT and protease inhibitors. Soluble fractions were isolated by centrifugation for 30 minutes at 100000 xg at 4°C. Aliquots (30 μl) of Glutathione-Sepharose (GE Healthcare) were equilibrated in lysis buffer and incubated with extracts (1 mg total protein) overexpressing GST, GST-PDRG1 or the truncated forms for 1 hour at 4°C. The gel samples were washed three times with cold PBS by centrifugation at 3500 xg for 5 min at 4°C before addition of GST overexpressing extracts (5 mg), alone or in combination with those containing the MAT proteins of interest (500 μg), to avoid unspecific binding. Parallel experiments were also carried out using purified MATα2, MATβ or MAT II proteins (100 μg). The mixtures were incubated with the gel for one additional hour at 4°C, and after extensive washing, the gel was boiled in Laemmli buffer. The bound proteins were loaded into SDS-PAGE gels and electrotransferred for western blotting.

### Animal models and cell treatment

Male Wistar rats (200 g) were subjected to acute D-galactosamine intoxication for 48 hours and control and treated livers extracted, as described previously [17]. Additionally, liver samples of 9-week old Long Evans Cinnamon (LEC) and control Long Evans (LE) rats were also used [32]. All animals received standard diets *ad libitum* and were sacrificed using CO_2_ asphyxiation. The experiments included in this study were approved by the CSIC Bioethics Committee and carried out in full accordance with Spanish regulations (RD 53/2013) and the European Community guidelines (2010/63/EU) for the use of laboratory animals. Tissue extraction and preservation was carried out as described [17, 32]. Effects on *Pdrg1* mRNA half-life were analyzed by RTqPCR using control and 10 mM D-galactosamine-treated H35 cells (3 x 10^5^), in the presence or absence of 5 μg/ml actinomycin D (Sigma), as previously described [17].

### RNA isolation and real-time RT-PCR

RNA purification and analysis was carried out as previously described using 100–150 mg of rat tissues or H35 cells (4 x 10^5^ cells)[6, 32]. Gene specific primers for rat *Pdrg1* were designed using the program Primer Express 3.0 (Applied Biosystems, Foster City, CA, USA) with Tm values between 58–60°C (sense 5’-GACCTGGACACCAAGAGGAA-3’, antisense 5’-GGTGCTCCTGATCTTTCTGG-3’); *Mat1a* and *18s* primers were previously described [32]. Reverse transcription and cDNA amplification were carried out as described [32], using 300 nM (*Mat1a* and *Pdrg1*) and 100 nM (*18s*) primer concentrations and Power SYBR Green PCR Master Mix (Applied Biosystems). Expression was evaluated using the ABI 7900HT Real-Time PCR system (Applied Biosystems) at the Genomic Service of our institute. Relative expression ratios were normalized to the geometric mean of the *18s* gene used as a control. Experimental efficiencies were calculated for each transcript and used to obtain the fold changes according to Pfaffl et al. [33].

### DNA methylation measurements

Genomic DNA was isolated from transiently transfected CHO cells (5 x 10^5^ cells) using the DNeasy kit (Qiagen) and the incorporation of methyl groups from [^3^H-methyl]-AdoMet (GE Healthcare) was followed by the inverse radioactivity assay described by Christman et al. [34] using *E*. *coli* SssI methylase (New England Biolabs).

### Silencing of Pdrg1 expression in H35 cells and production of stable clones

Reduction of rat *Pdrg1* expression was carried out by transfection of H35 cells (4 x 10^5^ cells) with SureSilencing shRNA plasmids (SaBioscience, IZASA, Madrid, Spain) containing sequences designed for this purpose (GGAGCACCTGGATAAAGAAAT, shRNA1; TCACCTTAAGACGAAGGAAAT, shRNA2; ACCTTAAGACGAAGGAAATGA, shRNA3; AGGAGCACCTGGATAAAGAAA, shRNA4) and a negative control sequence (GGAATCTCATTCGATGCATAC, CN). For initial evaluation of the silencing ability of each plasmid, transfections were carried out in triplicate for 48 hours, followed by enrichment of the transfected population with 1.8 mg/ml G418 (Gibco) for two weeks. RNA was extracted from half of the population and used for RTqPCR evaluation of *Pdrg1* expression. Only cells harboring plasmids inducing more than 70% reduction of *Pdrg1* expression at this point were used for further selection (S1 Fig). Stable clones for the negative control, shRNA3 and shRNA4 plasmids were finally obtained using 4 mg/ml G418 for two additional weeks. Approximately 200 stable clones for each plasmid were isolated and analyzed RTqPCR and one clone of each, the negative control (CN-10), shRNA3 (3–44) and shRNA4 (4–18), exhibiting reproducible behavior were selected for further experiments.

### Differential expression profile and microarray analysis

Four biological replicates of stable clones (CN-10, 3–44, 4–18) and a transiently transfected shRNA3 H35 enriched pool (shRNA3T), were independently hybridized for each transcriptomic comparison. Total RNA (200 ng) was amplified using One Color Low Input Quick Amp Labeling kit (Agilent Technologies, Santa Clara, CA USA) and purified with RNeasy Mini kit (Qiagen). Preparation of probes and hybridization was performed as described in the One-color Microarray Based Gene Expression Manual v6.5 (Agilent Technologies), using Rat Gene Expression Microarray v3 Agilent 4x44K. Briefly, for each hybridization 600 ng of Cy3 probes were mixed and added to 10x Blocking Agent (5 μl), 25x Fragmentation Buffer (1 μl) and Nuclease free water in a 25 μl reaction, incubated at 60°C for 30 minutes to fragment RNA and stopped with 2x Hybridization Buffer (25 μl). Samples were placed on ice and immediately loaded onto arrays, hybridized for 17 hours at 65°C and washed for 1 minute sequentially with GE wash buffers 1 and 2 at room temperature. Arrays were dried by centrifugation, images captured with an Agilent Microarray Scanner and spots quantified using Feature Extraction Software (Agilent Technologies). Background correction and normalization of expression data were performed using LIMMA [35, 36]. Linear model methods were used to determine differentially expressed genes. Each probe was tested for changes in expression over replicates by using an empirical Bayes moderated t-statistic [35]. Control of false discovery rate was achieved by correction of p-values as previously described [37]. The expected false discovery rate was controlled to be less than 5%. Hybridizations and statistical analysis were performed at the Genomics Facility of the Centro Nacional de Biotecnología (CNB-CSIC). FIESTA Viewer v1.0 was used to identify 114 genes exhibiting changes ≥2-fold with FDR<0.05, clustering and Heatmaps were prepared using Cluster [38] and Java TreeView [39], whereas pathway analysis was carried out with BioProfiling [40]. Verification of expression changes was carried out by RTqPCR for selected genes using RNA samples of stable clones and appropriate TaqMan probes (Table 3). Microarray results have been deposited in the GEO Database and are accessible through the series accession number GSE69337 (http://www.ncbi.nlm.nih.gov/geo/query/acc.cgi?acc=GSE69337).

**Table 3:** Data of TaqMan probes.

gene	reference	Overlapping exons
*Aacs*	Rn00675033_ml	16–17
*Acadm*	Rn00566390_ml	2–3
*Adm*	Rn01507680_gl	3–4
*Aldob*	Rn01768292_ml	8–9
*Hmgcs2*	Rn00597339_ml	5–6
*Lipc*	Rn01530834_ml	1–2
*Mat1a*	Rn00563454_ml	1–2
*Mat2a*	Rn01643368_gl	1–2
*Pdrg1*	Rn01535663_ml	3–4
*Sstr2*	Rn01464950_gl	1–2

### Growth curves

Eight replicas per experiment of each, H35 cells and stable clones (CN-10 and 4–18), were seeded (10000 cells/well) and grown for up to 8 days using the standard medium. At the desired time points the cell number was measured using crystal violet by the procedure of Gillies et al., as previously described [41, 42]. Briefly, wells were washed with PBS, cells fixed using 1% (v/v) glutaraldehyde (Fluka, Madrid, Spain) for 15 minutes and stained using 0.1% (w/v) crystal violet (Merck, Darmstadt, Germany) for 30 minutes. Following extensive washing, cells were allowed to dry for 24 hours, the color was solubilized with 10% (v/v) acetic acid (Merck) and the A_590_ measured.

### PDRG1 structural model

The rat PDRG1 sequence and the online Protein Homology/analogY Recognition Engine (PHYRE; http://www.sbg.bio.ic.ac.uk/~phyre/) were used to generate a structural model, which includes residues 27–106 of the protein. This information was employed for the design of PDRG1 truncated forms. Figures were prepared using PyMol (DeLano Scientific LLC, San Carlos, CA, USA).

### Determinations of enzymatic activities and protein concentrations

MAT activity was measured in column fractions (100–160 μl) of the different purification steps as previously described [23]. Additionally, this same activity was evaluated in the pooled peaks containing purified MATα1/GST-PDRG1 or MATα2/GST-PDRG1 complexes (160 μl). Assays including PDRG1 or histone II A (Sigma) were carried out after preincubation on ice for 5 minutes with either purified recombinant MATα1, MATα2 or MAT II oligomers (0.7 μM), using subunit molar ratios between 1:0 and 1:8 MATα/PDRG1 (160 μl) in a final reaction volume of 250 μl. LDH activity was determined spectrophotometrically in both cytosolic and nuclear fractions as previously described [6]. Protein concentrations were measured using the Bio-Rad protein assay kit (Bio-Rad) and bovine serum albumin as standard.

### Statistical analysis

GraphPad Prism v. 5.0 (GraphPad Software, La Jolla, CA, USA) was used for statistical analysis of the data, unless otherwise specified. Student’s t-test was preferred for analysis of experiments containing two groups, whereas one-way ANOVA with Bonferroni post-hoc test was utilized for multiple comparisons; differences were considered significant when p≤0.05.

## Results

### PDRG1 interacts with methionine adenosyltransferase α1

**Fig 1:**
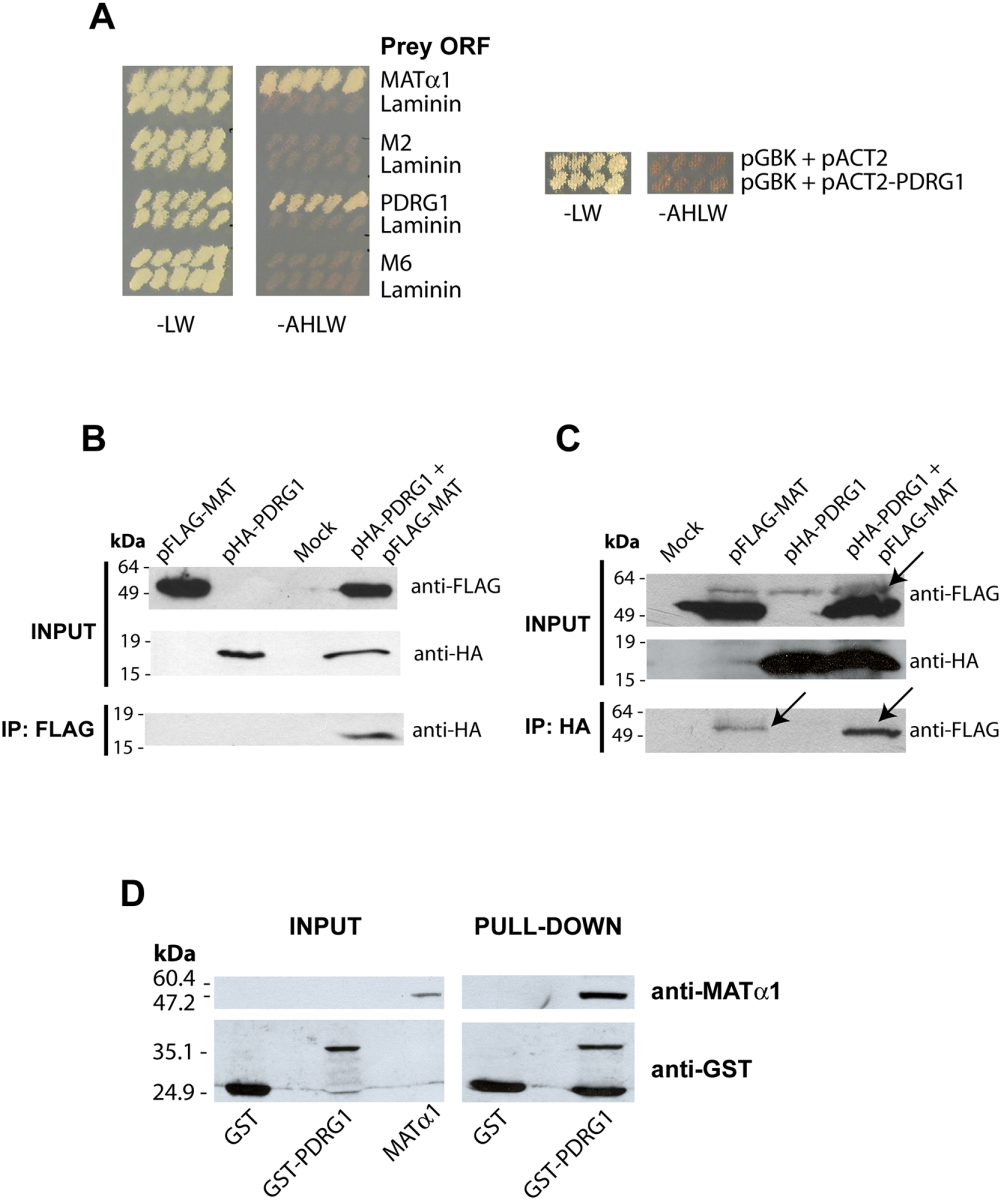
PDRG1 interacts with methionine adenosyltransferase α1. **(A)** Growth of yeast cotransfectants harboring pGBKT7-MATα1 (bait) and pACT2 plasmids (prey) including ORFs of MATα1, PDRG1, clone M2, clone M6 or laminin (negative control) in low (-LW) and high (-AHLW) stringency SC media. Additional controls including the empty pGBK plasmid are shown on the right. **(B)** Representative anti-FLAG immunoprecipitation results from four independent experiments using total lysates of CHO cells transiently cotransfected with pFLAG-MAT and pHA-PDRG1 or the empty plasmids (mock). The size of the standards is indicated on the left side of the panel. **(C)** Representative anti-HA immunoprecipitation data from three independent experiments utilizing total lysates of HEK 293T cells transiently cotransfected with pFLAG-MAT and pHA-PDRG1 or the empty plasmids (mock). Western blots of the input fractions were developed using anti-FLAG and anti-HA, whereas immunoprecipitates were analyzed using anti-HA or anti-FLAG with mouse TrueBlot ULTRA, as required. The arrow indicates an unspecific band recognized by anti-FLAG slightly over the FLAG-MATα1 signal in HEK 293T samples. The size of the standards is indicated on the left side of the panel. **(D)** Pull-down confirmation of the interaction using glutathione Sepharose beads loaded with GST or GST-PDRG1 and incubated with recombinant MATα1 plus excess GST. Results shown correspond to a typical experiments out of the five carried out; input fractions of the recombinant proteins used (left) and pull-down results (right) are shown. The size of the standards is indicated on the left side of the panel.

In order to identify proteins that interact with MATα1 a yeast two-hybrid screening was performed using a rat liver cDNA library and the full-length ORF of rat *Mat1a* as bait. Only one prey was further confirmed using high stringency conditions, its sequence corresponding to p53 and DNA damage-regulated gene 1 (*Pdrg1*; NM_001014762)(Fig 1A). Additional validation of the interaction was obtained using total lysates of CHO and HEK-293T cells transiently cotransfected with both pFLAG-MAT and pHA-PDRG1. Immunoprecipitation with anti-FLAG followed by western blotting using anti-HA revealed a band showing the expected size for the HA-PDRG1 fusion protein (155 amino acids; 17.9 kDa) only in cotransfected cells (Fig 1B). The inverse immunoprecipitation was also carried out using anti-HA followed by western blotting utilizing anti-FLAG and mouse TrueBlot ULTRA, to avoid hindrance of the FLAG-MATα1 band by the immunoglobulin heavy chains. Anti-FLAG detected an unspecific band with slightly slower mobility than FLAG-MATα1 in all the immunoprecipitates, whereas only cotransfected cells exhibited a band of ~50 kDa as expected for the FLAG-MATα1 protein (Fig 1C). Additionally, pull-down experiments were performed using glutathione Sepharose beads loaded with GST (~25 kDa) or GST-PDRG1 (~41 kDa) and *E*. *coli* extracts overexpressing MATα1. The presence of MATα1 among the proteins retained onto GST-PDRG1 beads was confirmed by western blotting using anti-MATα1 (Fig 1D).

### Interaction of MATα1 and PDRG1 involves the core structure of this protein

**Fig 2:**
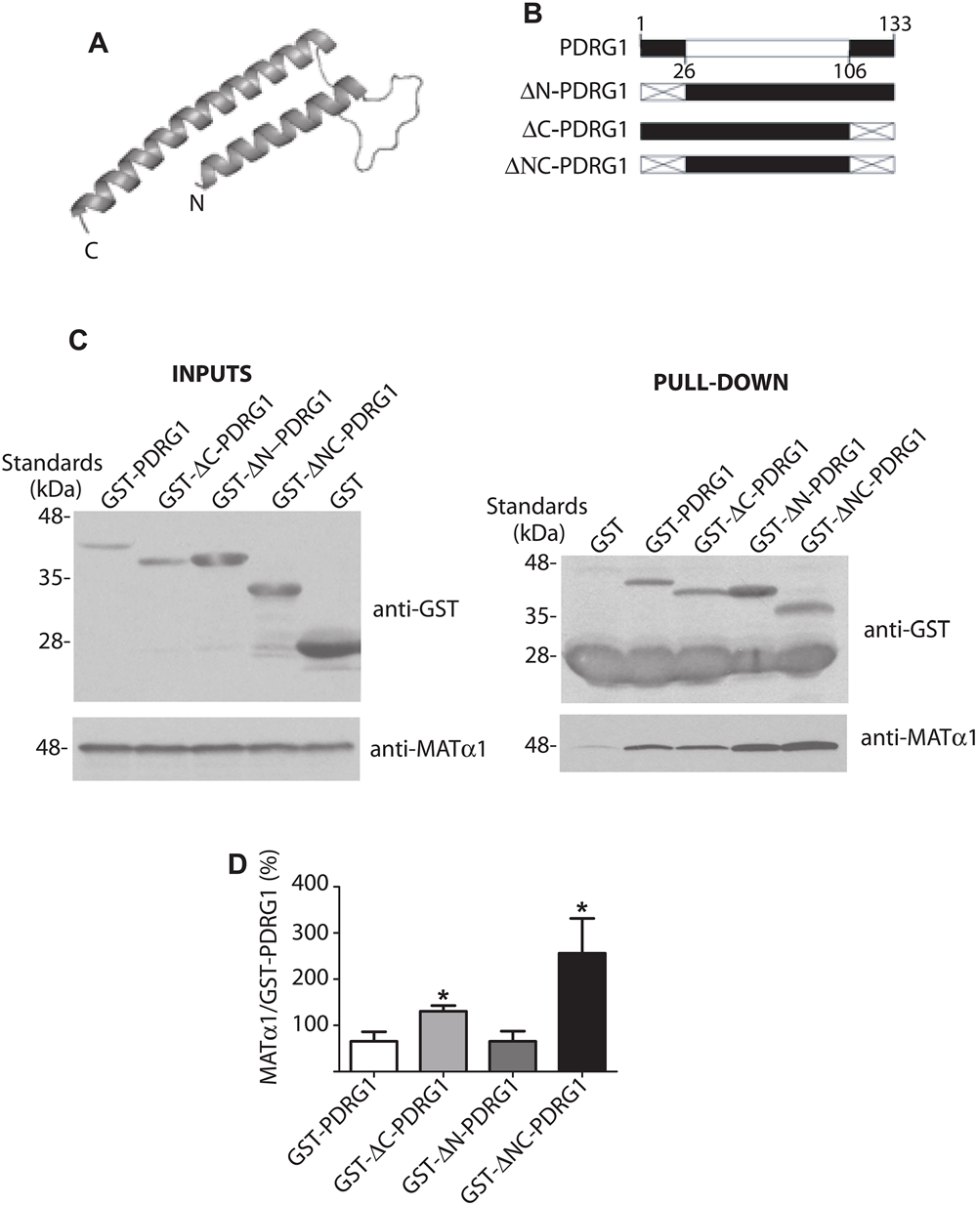
Structural model of rat PDRG1 and interaction of PDRG1 truncated forms with MATα1. **(A)** PDRG1 structural model comprising residues K27-Q106 obtained with PHYRE. **(B)** Schematic representation of PDRG1 and the truncated forms prepared; the modeled area (white box) and deleted sequences (crossed box) are indicated. **(C)** Representative western blots of pull-down experiments carried out with recombinant truncated PDRG1 forms and MATα1 using anti-GST and anti-MATα1. Incubations with MATα1 were carried out in the presence of excess GST to avoid unspecific binding. The size of the standards is indicated on the left side of the panels. **(D)** Quantification of the MATα1/GST-PDRG1 signal ratio (mean ± SEM) from seven independent pull-down experiments (*p≤0.05 vs GST-PDRG1).

There is no available structural information about PDRG1 that could serve to get insight into the interaction domain. Using the PHYRE online engine, prefoldin was identified as the closest structural homologue for PDRG1 and this information aided to build a structural model comprising residues K27-Q106. The model excluded the N- and C-terminal ends of the protein, and showed the PDRG1 core as two α-helixes linked by a loop (Fig 2A). Based on these data, three truncated forms of GST-PDRG1 were generated lacking the N- or C-terminal ends or both (Fig 2B). Pull-down assays were then used to assay their ability to interact with MATα1 in comparison with GST-PDRG1 (Fig 2C). No significant change in binding was detected for the mutant lacking the N-terminal (GST-ΔN-PDRG1), whereas removal of the C-terminal end increased the amount of MATα1 bound to both GST-ΔC-PDRG1 and GST-ΔNC-PDRG1 proteins (Fig 2D). These results suggested that binding with MATα1 occurs through the structural core of PDRG1, in an area partially covered by its C-terminal end.

### PDRG1 is a nucleocytoplasmic protein that interacts with MATα1 in the nucleus

**Fig 3:**
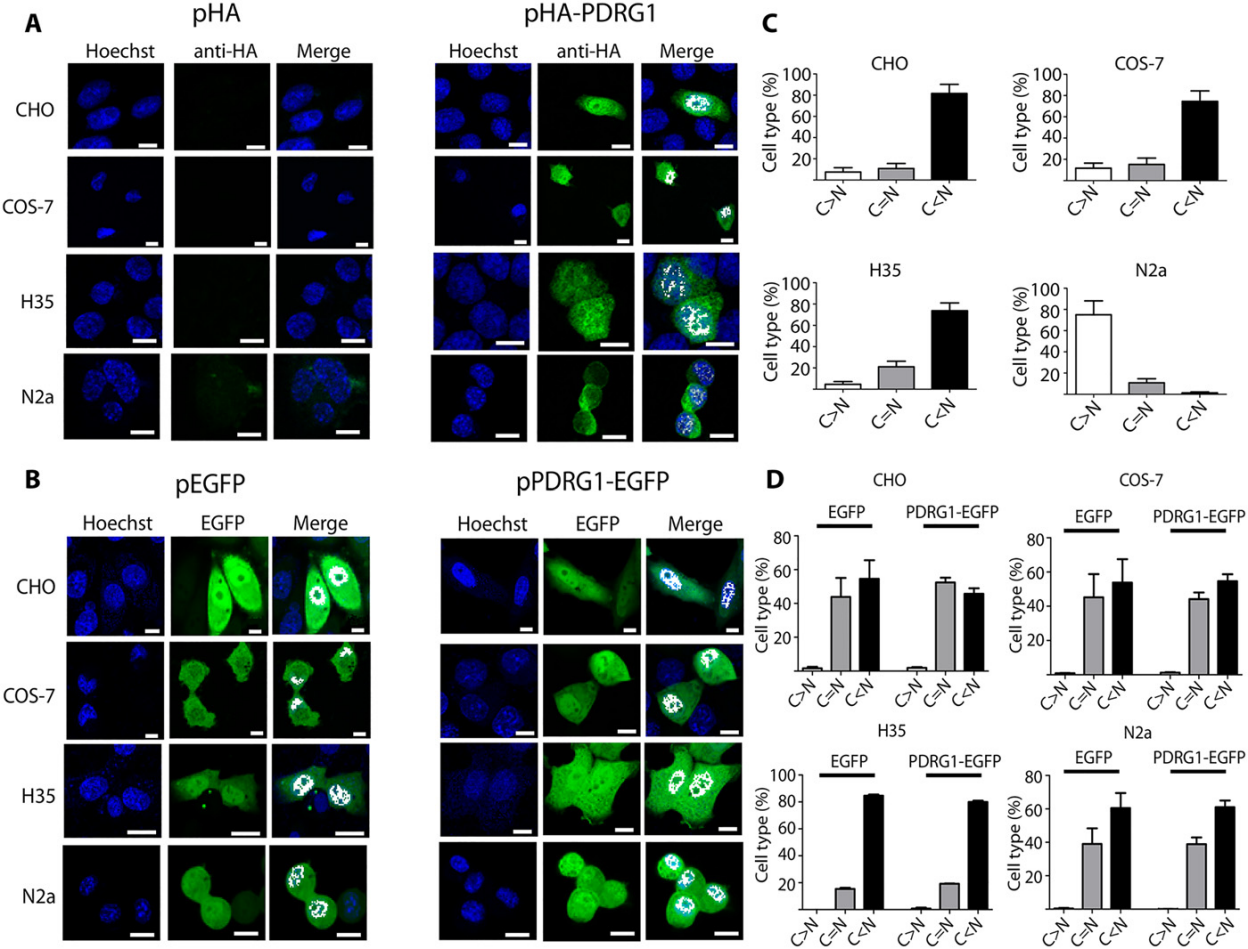
Subcellular localization of PDRG1 in mammalian cell lines. **(A)** Representative confocal immunofluorescence images of HA-PDRG1 localization using mouse anti-HA and anti-mouse Alexa Fluor 488; a minimum of three independent experiments were carried out in cuadruplicate. CHO (Chinese hamster ovary), COS-7 (monkey kidney), H35 (rat hepatoma) and N2a (mouse neuroblastoma) cells were transiently transfected with pHA, pHA-PDRG1, pEGFP or pPDRG1-EGFP. **(B)** Representative results of direct fluorescence localization using confocal microscopy of EGFP and PDRG1-EGFP; three independent experiments were performed in duplicate. Both panels show colocalization with Hoechst nuclear staining in white. **(C)** Histograms (mean ± SEM) show quantification results of nuclear (N) and cytoplasmic (C) fluorescence signals of a minimum of 200 cells per condition, using the Leica confocal software. Results of the C/N signal ratio calculated from immunofluorescence experiments are depicted. **(D)** Data of the C/N signal ratio from direct fluorescence observations. Cells were classified as: C>N with a ratio above 1.2; C = N when the ratio was 1 ± 0.2; and C<N with ratios below 0.8. Statistical evaluation was done by means of one-way ANOVA with Bonferroni post-hoc (*p<0.05 vs C = N). Scale bar = 10 μm.

**Fig 4:**
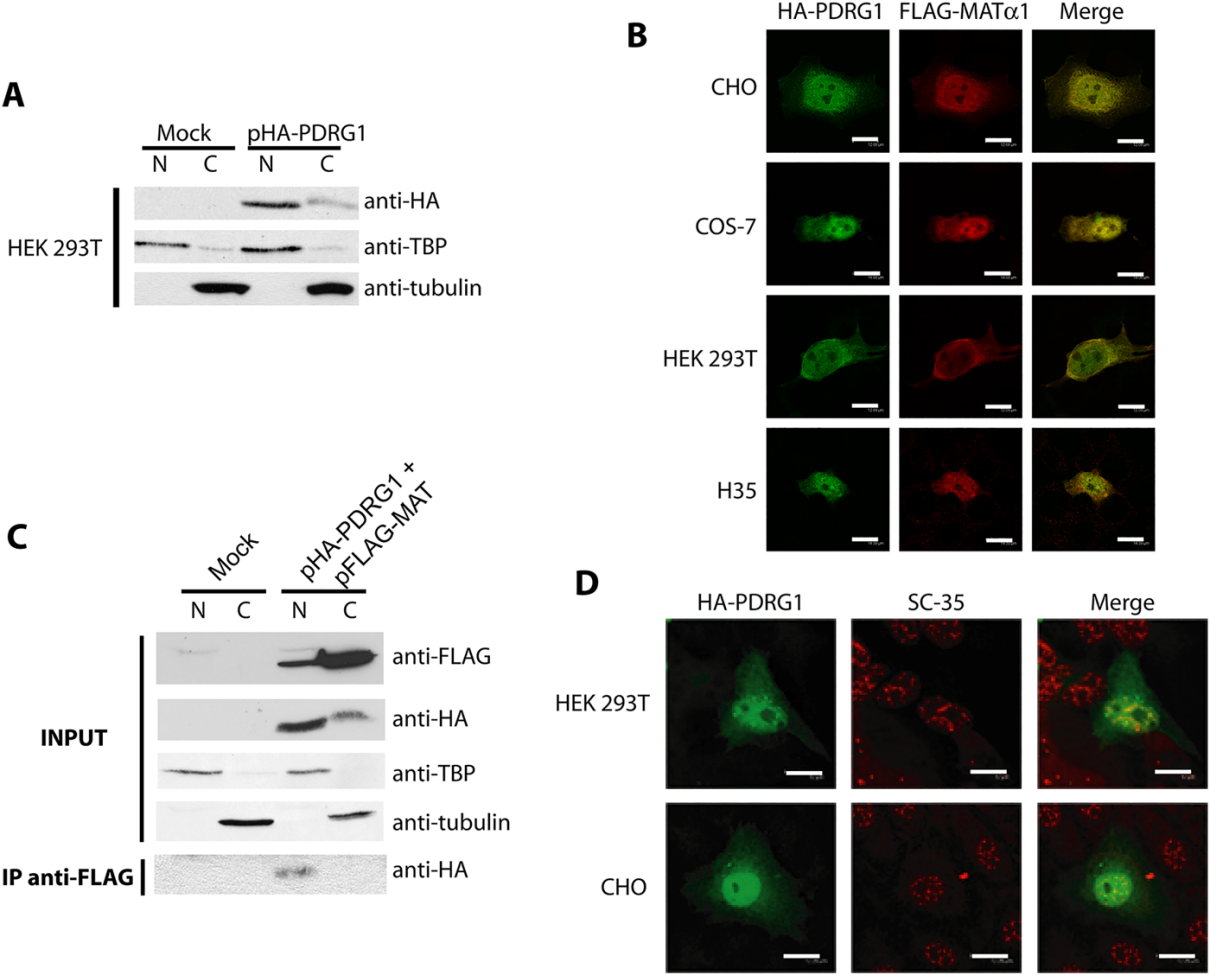
Subcellular distribution of HA-PDRG1 and the HA-PDRG1/ MATα1 interaction. **(A)** Representative western blots of nuclear (N) and cytoplasmic (C) fractions (50–70 μg) obtained from HEK 293T cells transiently transfected with pHA or pHA-PDRG1 in three independent experiments. Membranes were analyzed using anti-HA, anti-TBP (nuclear marker) and anti-tubulin (cytoplasmic marker). **(B)** Representative confocal immunofluorescence images (N = 50 per cell line) of CHO, COS-7, HEK 293T and H35 cells transiently cotransfected with pHA-PDRG1 and pFLAG-MAT obtained using mouse anti-HA, rabbit anti-MATα1 and the corresponding secondary antibodies coupled to Alexa Fluor 488 (green) or 546 (red). Colocalization of both proteins is shown in yellow (scale bar = 12 μm) **(C)** Representative results of anti-FLAG immunoprecipitations carried out in N and C fractions obtained from HEK 293T cells transiently cotransfected with pFLAG-MAT and pHA-PDRG1 or the empty plasmids (mock). Input fractions were analyzed using anti-FLAG, anti-HA, and antibodies against nuclear and cytoplasmic markers, whereas immunoprecipitates were examined using anti-HA. Results correspond to a minimum of three independent experiments. **(D)** Representative confocal immunofluorescence images of nuclear matrix preparations of HEK 293T and CHO cells overexpressing HA-PDRG1 using rat anti-HA (green), mouse anti-SC-35 (red) and appropriate secondary antibodies conjugated to Alexa Fluor dyes (N = 50 per cell line); colocalization appears in orange. Scale bar = 12 μm.

MATα1 is located mainly in the cytoplasm of hepatocytes, small amounts being detected in the nucleus, which is its preferred location in extrahepatic tissues and hepatoma cells [6]. On the other hand, PDRG1 was initially found as cytoplasmic aggregates using fixed NIH3T3 and HCT116 cells, but later identified in nuclear interaction complexes in LNCaP prostate cells [43, 44]. These data suggested different subcellular localizations for PDRG1 according to the cell type, which may differ from those of MATα1. Hence, we used confocal microscopy and the EGFP- and HA-tagged proteins to analyze PDRG1 distribution in additional cell lines, including hepatic cells (Fig 3). Both direct fluorescence and immunofluorescence showed the same subcellular distribution pattern with nuclear and cytoplasmic PDRG1 localization in all the cell lines examined (Fig 3A and 3B). However, quantification of the fluorescence signals demonstrated higher levels in the nucleus than in the cytoplasm in all the cases, except for N2a fixed cells (Fig 3C), and no statistical difference between PDRG1-EGFP and control EGFP distribution (Fig 3D). Confirmation of HA-PDRG1 localization to both subcellular compartments was also obtained by subcellular fractionation of transiently transfected HEK 293T cells (Fig 4A).

Confocal microscopy also demonstrated HA-PDRG1 and FLAG-MATα1 colocalization in both the cytoplasm and the nucleus in all cell types examined (Fig 4B). However, colocalization is not synonymous of interaction, and hence transiently cotransfected COS-7 and HEK 293T cells were used for subcellular fractionation and immunoprecipitation (Fig 4C). Western blots of the nuclear and cytosolic input fractions showed expression of both HA-PDRG1 and FLAG-MATα1, whereas only anti-FLAG immunoprecipitates from nuclear fractions exhibited anti-HA signals with the expected HA-PDRG1 size (Fig 4C). These data suggested that the interaction occurred in the nuclear compartment, where confocal microscopy also showed HA-PDRG1 colocalization with the nuclear matrix marker SC-35 (Fig 4D), as previously reported for MATα1.

### PDRG1 interacts with MATα1 producing larger oligomers in nuclear fractions

**Fig 5:**
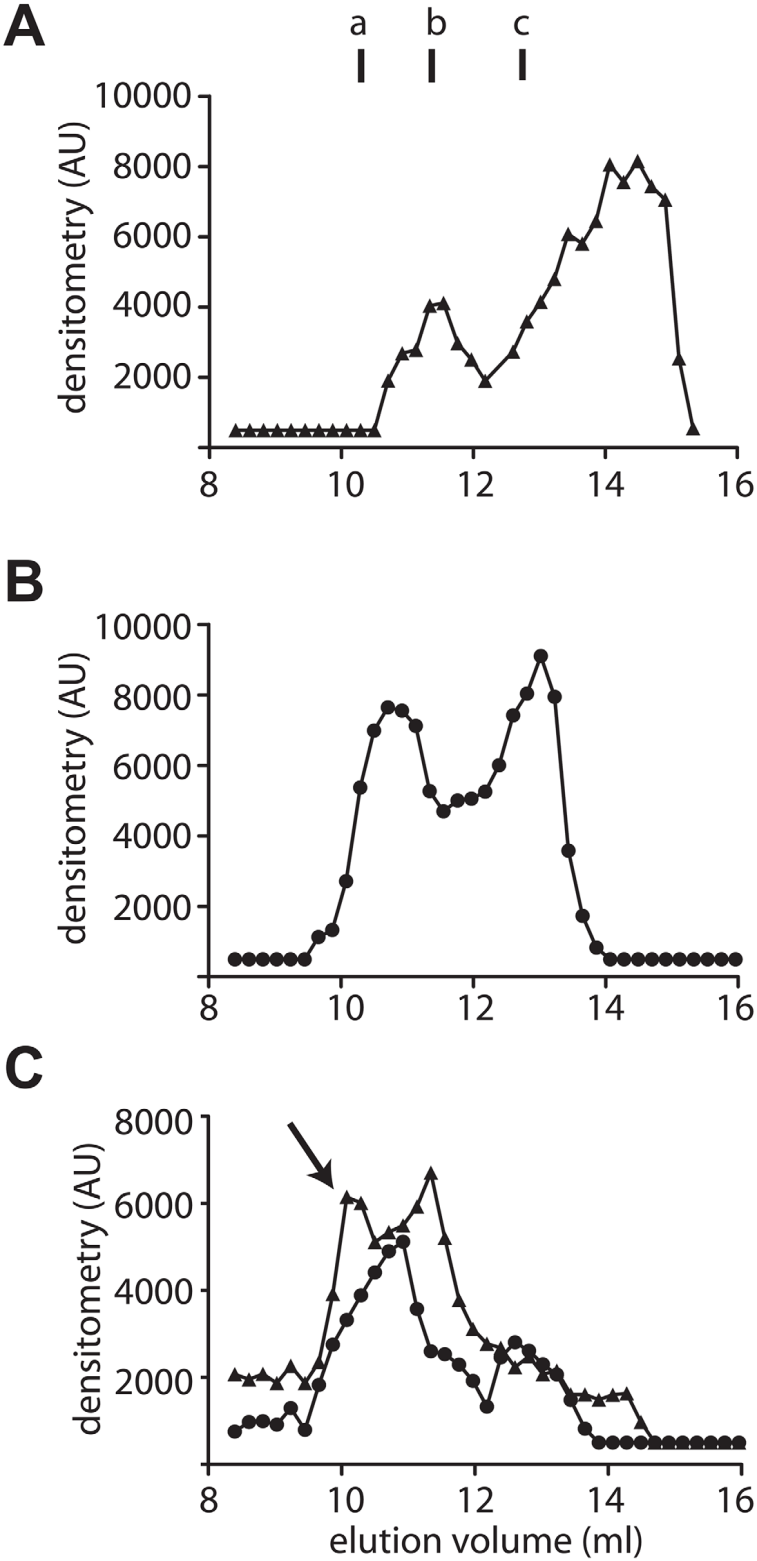
Evaluation of the PDRG1/ MATα1 association in nuclear extracts by analytical gel filtration chromatography. **(A)** Elution profile of nuclear extracts overexpressing HA-PDRG1 obtained on a Superose 12 10/300 GL column and analyzed by dot-blot using anti-HA. **(B)** Elution profile of nuclear FLAG-MATα1 detected using anti-MATα1. **(C)** Elution profile of nuclear extracts overexpressing HA-PDRG1 and FLAG-MATα1; the arrow indicates the new peak recognized by both antibodies (anti-HA (▲) and anti-MAT (●)). Elution of the protein standards was as follows: blue dextran (7.4 ml); ferritin (9.82 ml); β-amylase (a; 10.62 ml); aldolase (11.1 ml); alcohol dehydrogenase (b; 11.34 ml); conalbumin (c; 12.78 ml); ovalbumin (13.3 ml); carbonic anhydrase (14 ml); lysozyme (17.31 ml); and ATP (17.65 ml). The figure shows representative profiles obtained in five independent experiments.

MATα1 subunits associate into homo-tetramers (MAT I) and homodimers (MAT III) in the cytosol, whereas in the nucleus only MAT I and MATα1 monomers have been described [6, 17]. In order to explore whether PDRG1 displayed any preference for interaction with a specific MATα1 state, nuclear fractions from HEK 293T cells overexpressing HA-PDRG1, FLAG-MATα1 or both were analyzed by analytical gel filtration chromatography (AGFC)(Fig 5). Two elution peaks were detected for nuclear HA-PDRG1, one corresponding to a hexamer (11.55 ml) and another as predicted for a monomer (14.28 ml)(Fig 5A). Elution of nuclear FLAG-MATα1 occurred in the expected two peaks, corresponding to MAT I (10.71 ml) and MATα1 monomers (13.02 ml)(Fig 5B). A mixed profile was detected in nuclear fractions of cotransfected cells, as a result of the combination of peaks corresponding to HA-PDRG1 or FLAG-MATα1 homo-oligomers, in addition to a new peak eluting at 10.08 ml (Fig 5C). Both anti-HA and anti-FLAG antibodies detected this new peak, hence indicating the presence of the two proteins in a larger association state with an estimated molecular mass of 360 kDa, according to the elution profile of the standards.

### Tissular expression of *Pdrg1* and *Mat1a* showed different patterns

**Fig 6:**
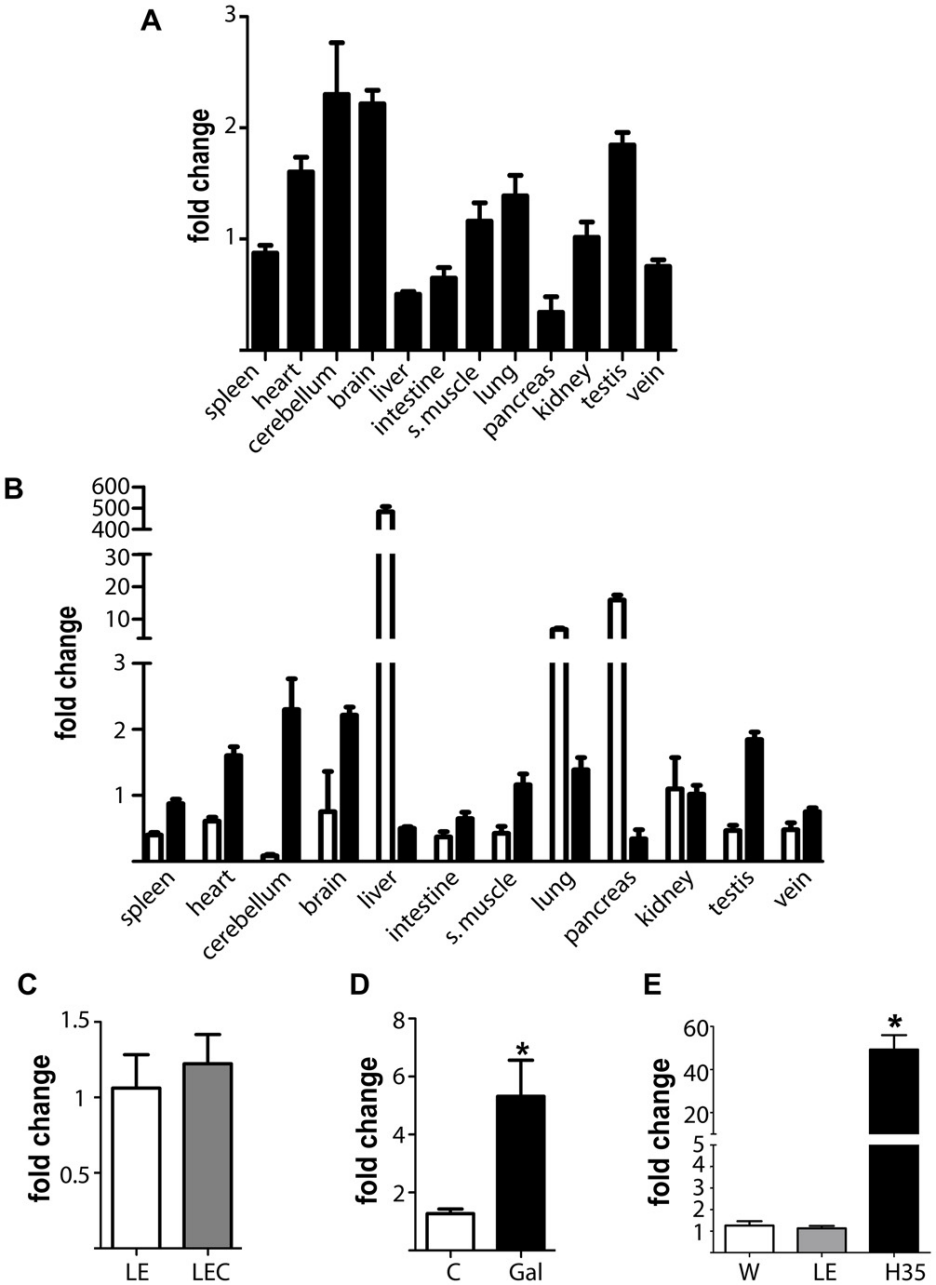
*Pdrg1* expression evaluated by real-time RT-PCR in rat tissues and models of hepatic disease. **(A)** *Pdrg1* expression levels in several rat tissues (N = 3), using kidney levels as reference for graphical purposes. **(B)** Comparison of *Mat1a* (white) and *Pdrg1* (black) expression levels using kidney levels as reference for graphical purposes. **(C)** Changes in *Pdrg1* hepatic expression at early stages of Wilson’s disease using Long Evans Cinnamon rats 9-weeks old (LEC, N = 6) and matched control Long Evans rats (LE, N = 5). **(D)** Changes in *Pdrg1* hepatic expression upon D-galactosamine intoxication for 48 h (control group N = 13, galactosamine group N = 11). **(E)** *Pdrg1* expression differences between rat hepatoma H35 cells (N = 12) and normal livers of Wistar (W; N = 13) and LE (N = 5) rats. Histograms show the mean ± SEM of the fold change calculated against the control group using *18s* data as reference. Statistical evaluation of the change in the animal models was performed by Students t-test against the appropriate control group (*p≤0.05).

The fact that PDRG1 immunoprecipitates with nuclear MATα1, suggested that this interaction may be more relevant in extrahepatic tissues or in hepatic disease, two environments in which MATα1 accumulates into this subcellular compartment [6, 17]. Therefore, we next examined whether *Pdrg1* expression followed the same trend than *Mat1a* using real-time RT-PCR (RTqPCR). All the rat tissues examined showed *Pdrg1* expression (Fig 6A), the highest levels being detected in cerebellum and brain, whereas the lowest were found in liver and pancreas. Surprisingly, this expression pattern was almost opposite to that exhibited by *Mat1a* (Fig 6B) and closer to the trend described for *Mat2a* and *Mat2b*.

We next examined hepatic *Pdrg1* expression in two animal models of liver disease and in hepatoma cells. Livers of 9-week old LEC rats, a model of Wilson disease, exhibited a moderate decrease in *Mat1a* expression (~20%) that was not followed by changes in *Pdrg1* expression as compared to the controls (Fig 6C). In contrast, livers of D-galactosamine intoxication (a model of acute liver injury) and H35 cells with strong reductions in *Mat1a* expression (~70% and >95%, respectively) exhibited 5- and 50-fold increases in *Pdrg1* mRNA levels, respectively, as compared to normal liver (Fig 6D and 6E). Putative effects on *Pdrg1* mRNA stability were also examined in H35 cells treated with D-galactosamine, where a trend towards increased half-life was detected in cells treated with actinomycin D and the drug (18.49 ± 5.18 vs. 39.19 ± 12.11 hours, p = 0.087). Protein levels were examined in hepatic subcellular fractions of control and D-galactosamine-treated rats using the available anti-PDRG1 antibodies. Given the low hepatic levels of the protein and the very low affinity exhibited by the antibodies, only extensive exposure of the membranes on ultrasensitive films showed a band of the expected size in the nuclear fractions (S2 Fig). Densitometric scanning confirmed a 3-fold elevation of nuclear PDRG1 levels in D-galactosamine intoxication. Thus, both proteins increase their nuclear levels in acute liver injury.

### PDRG1 was also an interaction target for MATα2

**Fig 7:**
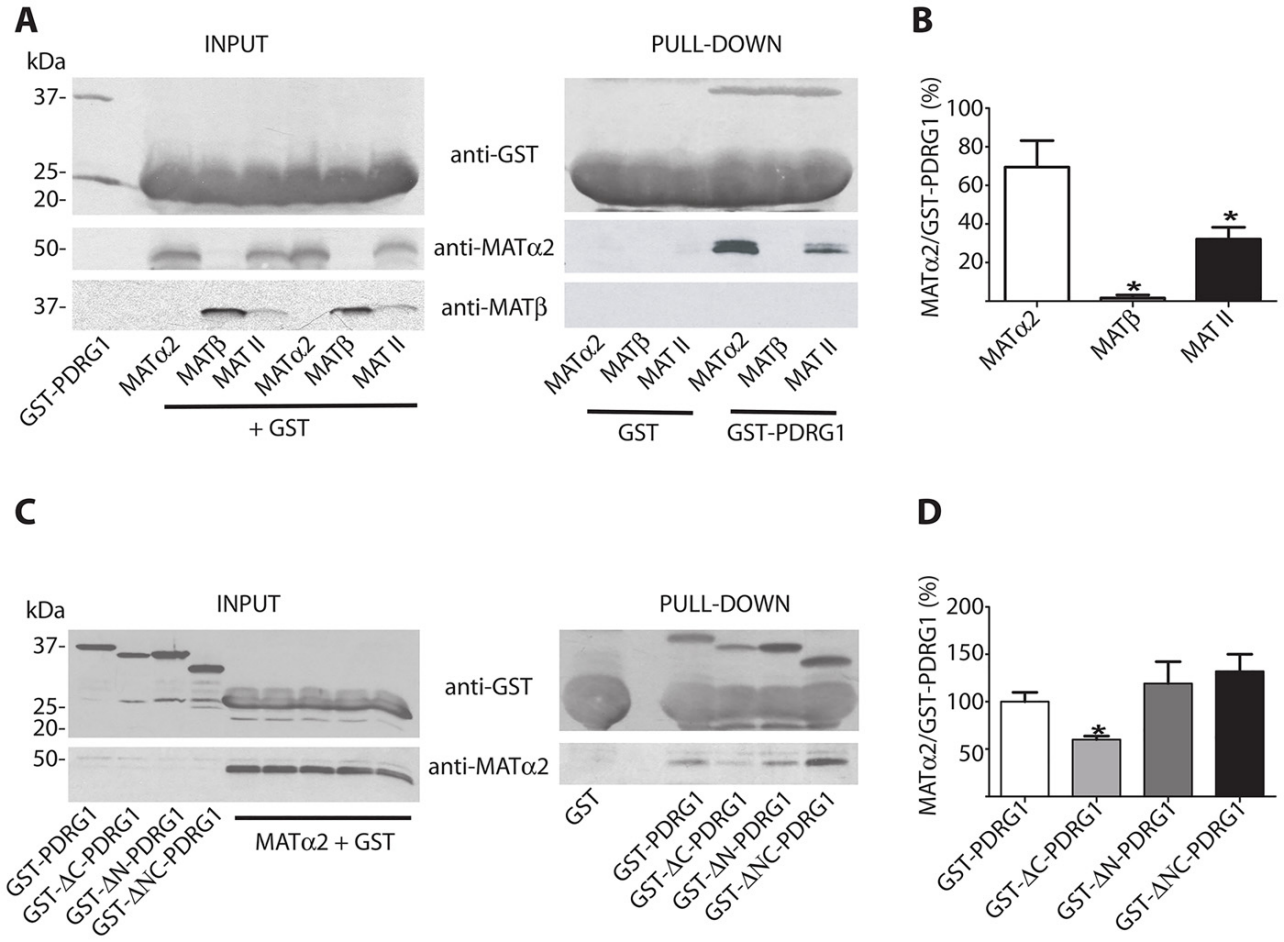
Pull-down analysis of PDRG1 interaction with MATα2 and MAT II. **(A)** Representative western blots of pull-down experiments using glutathione Sepharose beads loaded with GST or GST-PDRG1 and recombinant MATα2, MATβ or the hetero-oligomer MAT II; anti-GST, anti-MATα2 and MATβ were used for detection. The size of the standards is indicated on the left side of the panels. **(B)** Quantification of the MATα2/GST-PDRG1 signal ratio (mean ± SEM) from five independent pull-down experiments. **(C)** Representative western blots of pull-down experiments carried out with the truncated PDRG1 forms and recombinant MATα2 using anti-GST and anti-MATα2. The size of the standards is indicated on the left side of the panels. **(D)** Quantification of the MATα2/GST-PDRG1 signal ratio (mean ± SEM) from five independent pull-down experiments. All the incubations with MAT subunits or MAT II were carried out in the presence of excess GST to avoid unspecific binding. (*p≤0.05 vs GST-PDRG1).

The fact that *Pdrg1* and *Mat2a* share similar expression patterns, together with the high level of identity between MATα1 and MATα2, prompted us to examine the possibility that PDRG1 was also an interaction target for the later. The putative interaction was analyzed both in the absence or presence of MATβ using the human MAT II subunits, which exhibit >95% identity to their rat homologues. Pull-down experiments showed no interaction between GST-PDRG1 and MATβ, whereas MATα2 was able to bind to GST-PDRG1 both in the absence or presence of the regulatory subunit (Fig 7A). However, when the MAT II oligomer was formed the amount of MATα2 obtained by pull-down was reduced, according to the densitometric scanning of the data, and no β-subunit was detected (Fig 7B). The interaction with MATα2 was further analyzed using the truncated forms of GST-PDRG1 generated in the present study. Similarly to MATα1 elimination of the PDRG1 C-terminal end affected the interaction (Fig 7C), but in this case MATα2 binding was significantly reduced as deduced from data quantification (Fig 7D).

### Interaction of PDRG1 with MATα1 alters DNA methylation

**Fig 8:**
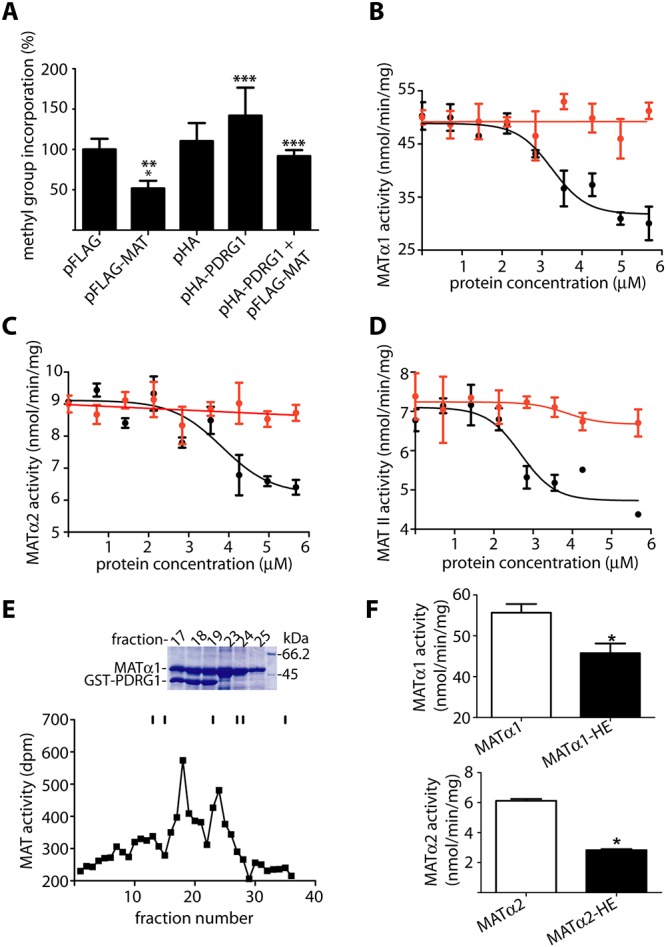
Effects of PDRG1 in DNA methylation and MAT activity. **(A)** Global DNA methylation levels of CHO cells transiently transfected with pHA-PDRG1, pFLAG-MAT or both plasmids evaluated with the inverse radioactive assay and compared to mock transfected cells. Incorporation of methyl groups into DNA (mean ± SEM) of five independent experiments carried out in triplicate is shown. For graphical purposes, the data are expressed as percentage of the pFLAG control taken as 100% (23392.65 ± 1790.07 cpm). Statistical analysis was performed using GraphPad Prism and changes were considered significant when p≤0.05 (*vs. pFLAG; ** vs. pHA; ***vs.FLAG-MAT). **(B)** Purified recombinant MATα1 (0.7 μM) was incubated with 0–5.6 μM PDRG1 (black) and S-adenosylmethionine synthesis determined; the panel shows results (mean ± SEM) of a typical experiment out of five carried out in triplicate. Controls including MATα1 and histone IIA (red) were also performed **(C)** Results (mean ± SEM) of a typical activity assay out of three performed in triplicate using MATα2 (0.7 μM). **(D)** Effects of PDRG1 (mean ± SEM) on MAT II activity (0.7 μM) from a typical experiment out of three carried out in triplicate. **(E)** Typical profile of a Biogel A purification of the MATα1/GST-PDRG1 complex followed by MAT activity. Elution of the standards is indicated with sticks that correspond to: Blue dextran (40 ml); ferritin (48 ml); aldolase (69 ml); conalbumin (81 ml); ovalbumin (84 ml); and ATP (105 ml). The upper part of the panel shows a stained SDS-PAGE gel of the relevant fractions as indicated on the top; the molecular size of the markers shown in the last lane (right) is indicated next to the corresponding stained band. **(F)** Comparison of the MAT activity shown by the MATα1/GST-PDRG1 (MATα1-HE; top) and MATα2/GST-PDRG1 complexes (MATα2-HE; bottom) vs. MATα1 or MATα2 homo-oligomers as correspond. The results shown are mean ± SEM of three independent experiments; *p<0.05.

The next question to examine was whether the PDRG1-MAT interaction affected AdoMet production. However, the low sensitivity of the MAT activity assays and the small nuclear level of the proteins precluded a direct evaluation of this parameter in nuclear fractions from cell lines. Similarly, direct measurements of nuclear AdoMet levels required a long process to eliminate the main cytoplasmic component, during which hydrolysis together with nuclear pore exchange takes place. Therefore, the indirect approach provided by measurement of global DNA methylation was preferred, together with the use of CHO cells that allow better cotransfection levels. Mock transfected cells showed global DNA methylation levels that depend on the AdoMet produced by MATα2 homo-oligomers and MAT II (Fig 8A). These levels were not significantly altered by HA-PDRG1 overexpression according to the inverse radioactive assay, although a tendency towards decreased DNA methylation was observed in all the assays performed. In contrast, FLAG-MATα1 overexpression let to DNA hypermethylation as previously described, an effect that was precluded by coexpression with HA-PDRG1 (Fig 8A). Altogether these data suggested a putative effect of the interaction on MAT activity.

### Interaction with PDRG1 reduced S-adenosylmethionine production by MATs

In order to further evaluate the effects of the interaction on AdoMet synthesis by MATs, the activity of MAT homo-oligomers was analyzed in the presence of PDRG1 *in vitro* (Fig 8B). For this purpose, purified recombinant MATα1 and MATα2 homo-oligomers were obtained and their activity measured in the presence of increasing concentrations of PDRG1. Either type of homo-oligomer showed up to 50% reduction in AdoMet synthesis in the presence of PDRG1 (Fig 8B and 8C). The calculated IC_50_ values were 3.34 ± 0.50 μM and 3.79 ± 0.48 μM for MATα1 or MATα2 oligomers, respectively. Furthermore, PDRG1 also reduced AdoMet synthesis by the MAT II hetero-oligomer obtained using purified recombinant MATα2 and MATβ subunits (Fig 8D), the calculated IC_50_ value being 2.76 ± 0.22 μM in this case. Effects of a non-related protein of a similar size such as histone IIA on MAT activity were also analyzed, the presence of this protein having no significant effect on AdoMet synthesis by homo- or hetero-oligomers (Fig 8B–8D). Additionally, MATα1/GST-PDRG1 and MATα2/GST-PDRG1 complexes were prepared and purified, before MAT activity measurements (Fig 8E). Again, decreased production of AdoMet (40–50%) by the isolated complexes was detected as compared to MATα1 or MATα2 homo-oligomers (Fig 8F). Altogether these data showed a reduction of MAT activity in the presence of PDRG1.

### Differential expression patterns produced by *Pdrg1* silencing

**Fig 9:**
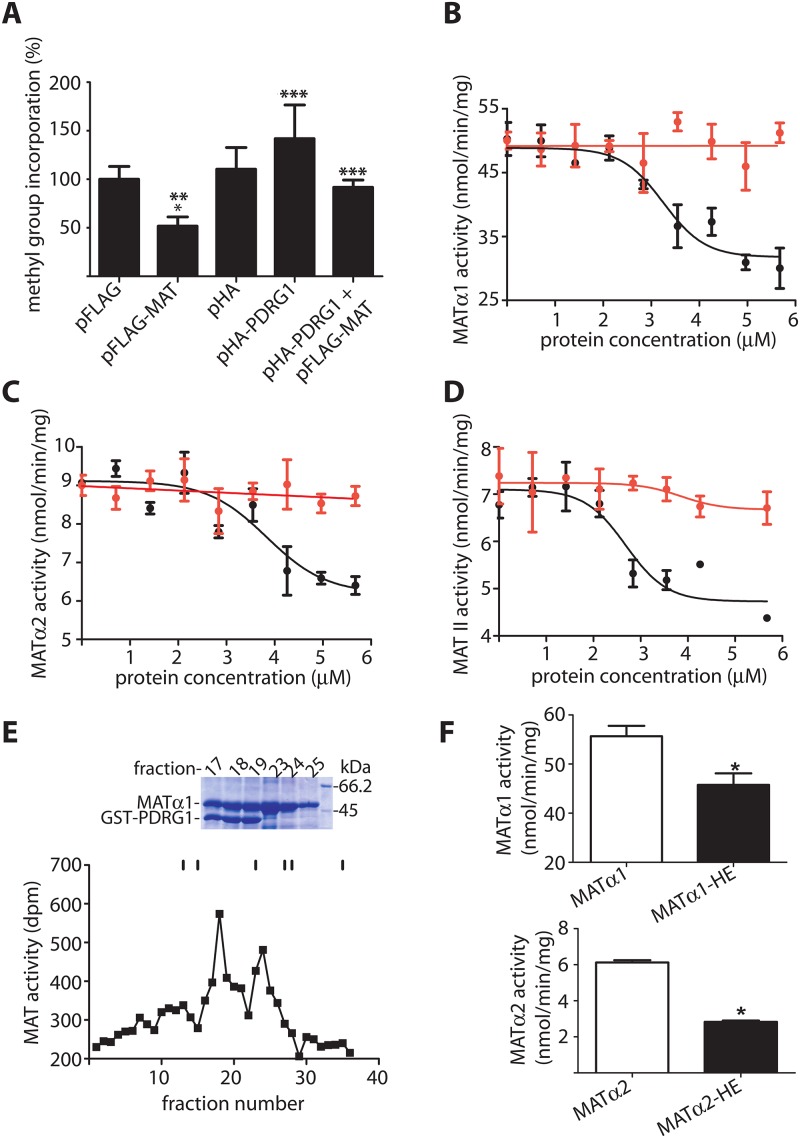
Differential expression analysis of *Pdrg1* silenced clones prepared in H35 cells. **(A)** Real-time RT-PCR analysis of *Pdrg1* expression using the *18s* gene as reference in the stable silenced clones (3–44 and 4–18) and the negative control clone (CN-10) prepared in H35 cells. The results shown are the mean ± SEM of four independent experiments carried out in triplicate. **(B)** Growth curves for H35 wild type cells (♦), the CN-10 (■) and 4–18 (●) clones; the figure shows the mean ± SEM of eight replicates of a representative independent experiment from the four carried out. **(C)** Pathway analysis of genes exhibiting expression changes ≥2-fold using Gene Ontology; only pathways with p<0.05 are indicated. **(D)** Real-time RT-PCR verification of expression changes (mean ± SEM; N = 4) in selected genes using the *Rn18s* gene as reference.

Silencing of *Pdrg1* expression will reduce PDRG1 levels and, in turn, decrease its possibilities of interaction with MATs and the indirect effects observed on DNA methylation. For this purpose, rat hepatoma H35 cells were chosen given that RTqPCR results demonstrated their elevated *Pdrg1* levels and that nuclear accumulation of MATα1 has been described in hepatoma cells. Therefore, H35 cells were transfected with appropriate shRNA plasmids against *Pdrg1* and stable clones isolated. Among those exhibiting reproducible behavior, clones CN-10 (negative control), 3–44 (shRNA3) and 4–18 (shRNA4) were selected for further analysis. *Pdrg1* expression was reduced by 50% and 70% in 3–44 and 4–18 clones, respectively, as compared to CN-10 (Fig 9A). Crystal violet assays did not detect alterations in cell growth for any of the stable clones, as compared to the wild type cell line (Fig 9B). RNAs of CN-10, 3–44 and 4–18 clones, as well as, RNA of an enriched pool of shRNA3 transiently transfected cells (shRNA3T) were used for expression analysis using microarrays. Genes exhibiting changes ≥2-fold with FDR<0.05, according to LIMMA analysis, were identified. Pathway analysis was performed with BioProfiling using data of 114 genes (74 upregulated and 40 downregulated) exhibiting similar behavior in the three silenced samples (S1 Table). The consistency of their behavior between biological replicates can be observed in the heatmap representation of the data (S3 Fig).

Only 93 genes of the input list (81.5%), most of them among those upregulated, were recognized and classified into the following GO pathways (p<0.05): response to starvation (6; p<0.001), lipid metabolic process (6; p = 0.01), liver development (5; p = 0.01), extracellular space (11; p = 0–01), cytoplasm (28; p = 0.01), response to glucocorticoid stimulus (5; p = 0.02), response to organic cyclic substance (6; p = 0.02) and extracellular region (13; p = 0.04) (Fig 9C and S4 Fig). Selected genes, at least two of pathways with p≤0.02, were used for verification of expression changes by RTqPCR, together with *Pdrg1*. Results were consistent with those of microarrays, in general, changes being larger in clone 4–18 than in clone 3–44, as compared to the negative control CN-10 (Fig 9D). Some GO pathways identified in microarray data were related to processes in which expression of *Mat* genes is altered, especially cancer development. In fact, *Sema3c*, *Id1*, *Cxcl1* and *Ctgf*, which are upregulated in a variety of cancer types, were downregulated upon *Pdrg1* silencing. In contrast, no relevant expression changes were detected in *Mat1a* and *Mat2a* during analysis of the microarray data, and only inconsistent and modest alterations (≤1.5 fold) were found by RTqPCR in the stable clones (S5 Fig). Therefore, the changes detected seem independent of alterations in the expression *Mat* genes.

## Discussion

Remodeling of epigenetic modifications is a process that continuously takes place during the life of an organism, in order to activate or repress the genes required for growth, the response to different insults, etc. AdoMet is among the substrates needed by the enzymes performing these modifications, and a reduction of its concentration is a common trait for a large variety of pathologies [3, 5, 14]. Moreover, results derived from mouse models showing both low (*Mat1a*^-/-^) and high (*Gnmt*^-/-^) AdoMet levels suggest the existence of a safe range of hepatic concentrations for this metabolite to maintain normal growth [45, 46]. Thus, it is important to know the mechanisms and actors involved in AdoMet homeostasis, which are been mainly studied in liver. The results obtained to date using models of hepatic disease show that altered concentrations of this metabolite commonly derive from the *Mat1a*/*Mat2a* expression switch, and post-translational modifications on cytosolic MATα1 induced by nitrosative and oxidative stress [15, 17, 21–23, 32, 47]. Identification of the interaction between PDRG1 and MATα1, together with the reduced MAT activity exhibited by the three isoenzymes in the presence of the former, now adds a new actor into the mechanisms that control methylation levels. In fact, PDRG1 becomes, together with MATβ [48–50], the only two interaction targets known for MATα1.

The reduced information available regarding PDRG1 raises doubts about where and how the MATα1-PDRG1 interaction takes place. First, commercial Northern blots of normal human tissues show the testis as the tissue exhibiting the highest levels of *PDRG1* expression [43]. This fact is now confirmed by RTqPCR using normal rat tissues, although similar expression levels are also detected in rat brain and cerebellum. In contrast, rat tissues exhibiting high levels of *Mat1a* expression, especially the liver, but also pancreas and lung, display the lowest expression levels for *Pdrg1*, a result that can be also inferred from human data [43]. Actually, the *Pdrg1* expression pattern matches that of *Mat2a* and seems opposite to that of *Mat1a* in normal tissues [6, 51]. This observation is further reinforced by detection of increased *Pdrg1* expression in D-galactosamine-treated livers, a model where elevated *Mat2a* expression together with decreased *Mat1a* mRNA levels was previously reported [17]. Second, the PDRG1 subcellular distribution is not clearly established [43, 44, 52], and hence may not match that of MATα1. Through the use a variety of cell lines of diverse origin, confocal microscopy and subcellular fractionation, we now demonstrate that PDRG1 is a nuclear and cytoplasmic protein, although a preference for nuclear localization is detected upon quantification of the data. These results confirm initial reports showing PDRG1 as a cytoplasmic protein [43], and also those in which the protein is found involved in nuclear protein-protein interactions [44, 52]. Furthermore, immunoprecipitation and AGFC results demonstrate that although MATα1 and PDRG1 colocalize in both subcellular compartments, their interaction only occurs in the nucleus, where both proteins also colocalize with the spliceosome marker protein SC-35. Interestingly, this result may be of special importance in extrahepatic tissues or in liver injury, two situations where MATα1 localizes or accumulates into the nuclear compartment, respectively [6, 17], thus enhancing the probability of interaction with PDRG1.

The presence of a helix-turn-helix motif and a β-prefoldin-like domain in PDRG1 were early identified [43], and this same sequence was also recognized by PHYRE to construct a structural model of rat PDRG1 that excludes approximately 26 residues from either end of the protein. Characterization of recombinant PDRG1 shows its elution in a volume corresponding to a hexamer, an association state that has been previously attributed to prefoldins [53, 54]. The recombinant protein is able to interact with MATα1, leading to a larger association state according to AGFC data. Moreover, PDRG1 is also able to interact with MATα2, as expected from the *Mat2a* expression pattern and the high sequence conservation among MATα subunits [3, 4]. In both cases, the MATα-PDRG1 interaction is altered by deletion of the C-terminal end of PDRG1, a fact that could be anticipated since a sequence normally involved in protein-DNA or protein-protein interactions is removed [43]. Unexpectedly, this deletion increases MATα1-PDRG1 interaction, hence suggesting a role for the C-terminal in the control of this binding that seems to involve the core of the predicted structure. Such an arrangement should not interfere with the interaction site proposed for all prefoldin-like proteins (URI, Art27, PDRG1, PFD2 and PFD6) of the R2TP/prefoldin-like complex [44, 52, 55], and that involves the hook of the prefoldin-like domain [44]. Conversely, the fact that MATα1 and MATα2 interact with PDRG1 suggests the putative involvement of a common interaction motif in the MATα subunits, which in turn, may lay close or superimpose to the β-subunit binding site, according to results of pull-down experiments with MAT II, where no MATβ is recovered.

**Fig 10:**
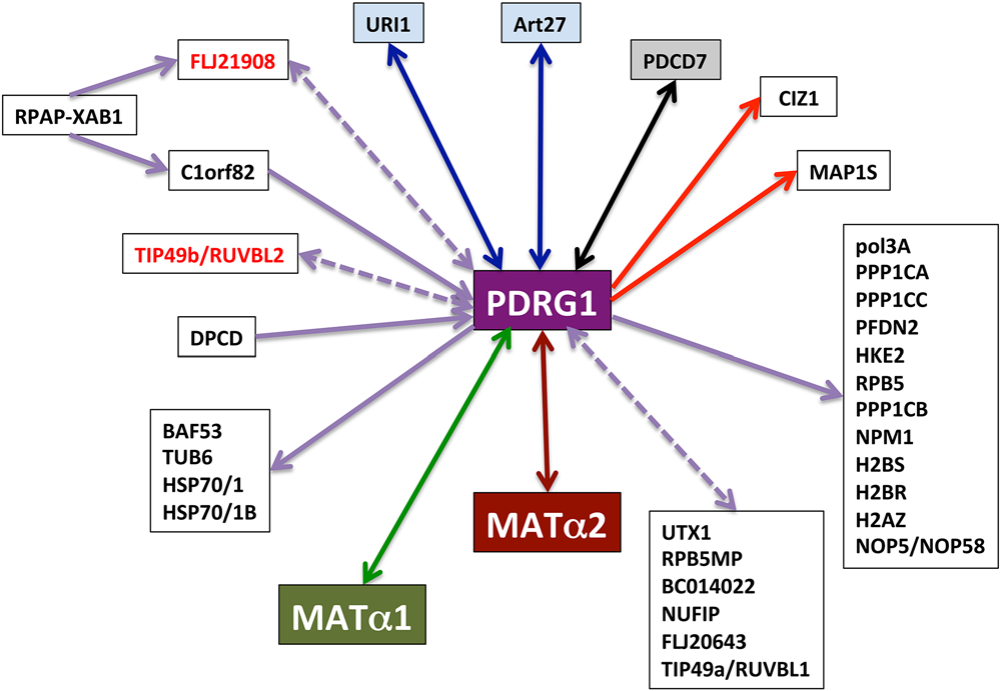
Schematic representation of PDRG1 interactions. PDRG1 interactions from the literature and the present work are presented. Arrows indicate interactions identified by a single method, bait (tail) and prey (head). Double-headed arrows indicate interactions confirmed by several methods (solid lines) or by the same method using either protein as bait (dashed lines). Proteins indicated in red have been identified in several studies by different authors. Arrow’s color codes indicate: yeast two-hybrid (YTH; red); affinity-purification coupled to mass spectrometry (AP-MS; violet); immunoprecipitation (IP) and YTH (black); YTH, IP, pull-down (PD) and activity (green); AP-MS and IP (blue); PD and activity (brown).

MATα subunits appear as two of the few validated targets for PDRG1, together with: i) PDCD7, a component of the U12-type spliceosome that is involved in the modulation of apoptosis [56]; and ii) URI and Art27/UXT, components of the prefolding complex [44](Fig 10). However, our data not only demonstrate the validity of the interaction, but also its putative role in the control of MAT activity. PDRG1 interaction with MATα1, MATα2 or MAT II oligomers reduces their ability to synthesize the methyl donor, thus indicating that the interaction takes place with their active oligomeric assemblies. Moreover, the data also suggest that *in vivo* the interaction may only involve the MAT I isoenzyme and the MATα2 dimer, according to the subcellular location of the MATα1-PDRG1 interaction (no MAT III is detected in the nucleus) and the pull-down results for MAT II. Furthermore, MATα1 overexpression is known to induce DNA hypermethylation in hepatoma cells [6], the preventive effect exerted by coexpression with PDRG1 confirming the relevance of the interaction for the control of nuclear methylations. CHO cells mainly express *Mat2a* and *Mat2b*, and in this context overexpression of PDRG1 alone has no significant effect on DNA methylation levels. Reasons for this lack of effect may rely in aspects favoring a tighter MATα2_2_-MATβ interaction or additional interactions [10, 18], which could preclude MATα2-PDRG1 interaction and the effects on DNA methylation. Additionally, aberrant DNA methylation is a well-established characteristic of cancer cells [57], and recent studies demonstrated global DNA hypomethylation correlating with enhanced expression of *PDRG1* in a variety of human non-hepatic tumors [56, 58, 59]. In our study, no morphological changes or apoptosis are detected upon PDRG1 overexpression, confirming previous reports [43]. However, stable clones with partial silencing of *Pdrg1* (up to 70%) show normal morphological characteristics and growth, in contrast to the severe effects on cell growth, invasion and increased apoptosis previously described upon PDRG1 depletion [56, 59]. This difference may rely on the use of diverse cell lines or silencing reactants and methods. In fact, apoptosis and impairment of cell growth may be the underlying cause for the failure to obtain stable clones with >70% downregulation of *Pdrg1*, regardless of the large number of clones examined in our study. The lack of significant changes in *Mat1a* or *Mat2a* expression in our silenced clones do not exclude the possibility that total suppression of *Pdrg1* expression enhances MAT levels and, in turn AdoMet concentrations, known to be pro-apoptotic in hepatoma cells [60].

Differential expression changes induced by *Pdrg1* silencing are consistent between stable clones and transient transfected pools. These modifications involve upregulation of GO pathways such as response to starvation, liver development, response to glucocorticoid stimulus or lipid metabolic process. Common genes shared by these pathways include: i) *Acadm* that is present in the four routes and encodes medium-chain specific acyl-CoA dehydrogenase, the enzyme catalyzing the first step of fatty acid β-oxidation [61]; ii) *Aacs*, *Aldob*, *Lipc* and *Hmgcs2* that are shared by three of these pathways and which codify for acetoacetyl-CoA synthetase, aldolase B, hepatic triglyceride lipase and 3-hydroxy-3-methylglutaryl-CoA synthase 2, respectively; and iii) *Adm* that is included in two of these pathways and which encodes adrenomedullin. Among them, the highest upregulation corresponds to *Aldob* (4–8 fold), followed by *Hmgcs2* (3–6 fold), thus suggesting a need to increase the glycolytic flux together with ketogenesis, which in turn provides lipid-derived energy during fasting. Additionally, genes normally upregulated in several types of cancer cells (*Sema3c*, *Id1*, *Cxcl1*, *Ctgf*) appear downregulated by *Pdrg1* silencing [62–65]. Therefore, the changes induced in hepatoma cells by *Pdrg1* downregulation seem to follow an opposite pattern than those exhibited by *Mat1a*^*-/-*^ or *Gnmt*^*-/-*^ livers with hepatic damage (esteatosis, hepatocellular carcinoma), which show induced or normal lipogenesis, respectively [45, 66–71]. Another characteristic of hepatocytes from these mice is their enhanced basal proliferation [67, 68, 72], but again no change in cell growth is detected in the *Pdrg1* silenced clones. Although the lack of data about *Pdrg1* expression in these mice precludes further comparison, altogether these data support a role for PDRG1 in the modulation of changes induced by variations in AdoMet concentrations.

The nuclear localization of the MATα1-PDRG1 interaction suggests that its effects may have special importance in that compartment, which is the preferred MATα1 site in extrahepatic tissues and in injured liver [6, 17]. These are two situations in which *Mat1a* expression is very low or dramatically reduced, hence suggesting a specific role for PDRG1 in the control of MAT activity by the remaining MAT I isoenzyme. Upregulated *Pdrg1* expression has been reported in UV-irradiated cell lines [43], in the presence of genotoxic agents [56] and in human tumors [56, 58, 59]. Now we also show that this upregulation occurs in H35 cells and acute liver injury, two conditions with different levels of *Mat1a* (minute or strongly reduced) and *Mat2a* expression (high and increased). Higher *Pdrg1* expression in these two environments does not lead to significant changes in global DNA methylation [6, 17], whereas either MATα1 overexpression or silencing of certain microRNAs induce DNA hypermethylation and increased nuclear levels of this protein [6, 73]. In contrast, coexpression of both MATα1 and PDRG1 precluded DNA hypermethylation, hence confirming the inhibitory role of the interaction and providing a clue to understand the lack of changes in this parameter observed in acute liver injury. Unexpectedly, both MATα1 overexpression in H35 cells [6] and liver injury [17] concur with increased levels of the me3K27H3 repression mark, despite the high expression levels of *Pdrg1* in these environments. A putative explanation may derive from results on miR-214 expression in cancer, where downregulation of this microRNA inversely correlates with *Pdrg1* expression [59], and accumulation of Polycomb Ezh2 methyltransferase is detected [74]. This methyltransferase is not only responsible of me3K27H3, but also controls DNA methylation through recruitment of DNA methyltransferases in the context of Polycomb repressive complexes 2 and 3 [75].

The possibility exists that in spite of nuclear accumulation of MATα1, the levels reached or the amount of MAT I formed, are not enough to cope with the AdoMet requirements of the normal cell to struggle against severe insults. In this context, oxidative stress may exert additional roles as previously hypothesized [25], among others: i) inhibiting MAT I/III [20, 24, 76]; ii) promoting anomalous subcellular distribution of MATα1 [17]; or iii) enhancing affinity between MAT II subunits [10]. Through this last option oxidative stress promotes production of the less active isoenzyme MAT II (with the lowest V_max_), probably precluding its interaction with PDRG1, a process that seems to require displacement of MATβ. Therefore, under conditions in which a larger supply of AdoMet is needed to accomplish the required epigenetic remodeling for response against an insult, interaction with PDRG1 may reduce this provision leading to either cell death or transformation. This last consequence may be favored by additional PDRG1 interactions involving the URI/prefoldin complex (i.e. during RNA polymerase II assembly)[44, 52, 55, 77]. Finally, altogether the results presented in this work made us hypothesize that the oncogenic role of PDRG1 may rely, at least in part, to its counteracting effect on repression of key genes for tumor progression through interaction with MAT I.

## Supporting Information

S1 Fig *Pdrg1* expression levels in transient transfections using shRNA plasmids. H35 cells were transiently transfected for 48 hours with shRNA plasmids harboring sequences for *Pdrg1* silencing (shRNA1-4) and a negative control (CN). Transfected cells were selected for 2 weeks with 1.8 mg/ml G418. Expression levels were analyzed by real-time RT-PCR using appropriate primers and SybrGreen. The figure shows results of a typical experiment of the three carried out in triplicate. Changes in *Pdrg1* expression (mean ± SEM) were calculated against H35 wild type cells and using the *18s* gene as reference. Statistical evaluation was performed by one-way ANOVA with Bonferroni post-hoc (*p≤0.05 vs. H35; ** p≤0.05 vs. CN).
(TIF)

S2 Fig PDRG1 levels in hepatic nuclear fractions of D-galactosamine intoxicated rats. Subcellular fractions of the whole liver from control (N = 4) and D-galactosamine (N = 6) intoxicated rats were analyzed by western blotting. Lamin B1 levels were used as reference, whereas tubulin signals were used to establish cross-contamination with cytosolic fractions. Bands with the PDRG1 expected size (~15 kDa) were only detected in nuclear fractions upon long exposure to ultrasensitive films. The size of the standards appears indicated on the left side of the anti-PDRG1 panel. Densitometric scanning of these signals was carried out to obtain the PDRG1/lamin B1 ratio (mean ± SEM; *p≤ 0.05).
(TIF)

S3 Fig Graphical representation of microarray differential expression changes. Microarray expression data were analyzed with FIESTA Viewer to identify genes exhibiting changes ≥2-fold with FDR<0.05. These data were used for clustering and preparation of heatmaps using Cluster and Java TreeView, respectively. The figure shows results (N = 4) of up- and down-regulated genes in stable clones CN-10, 3–44 and 4–18, as well as, in shRNA3T.
(PNG)

S4 Fig Heatmaps of microarray results for relevant pathways according to BioProfiling analysis. The figure highlights expression results of genes identified with BioProfiling (p≤0.01). The pathways including these genes are: Response to starvation (GO: 0042594); Lipid metabolic process (GO: 0006629); Liver development (GO:0001889); Extracellular space (GO: 0005615); Cytoplasm (GO: 0005737).
(TIF)

S5 Fig Expression of *Mat* genes evaluated by real-time RT-PCR in stable clones. CN-10, 3-44-and 4–18 stable clones (N = 4) were used to analyze putative effects of *Pdrg1* silencing on *Mat1a* and *Mat2a* expression using RTqPCR and appropriate TaqMan probes. The figure shows the mean ± SEM of measurements carried out in triplicate, where expression changes were calculated against CN-10 and using the *18s* gene as reference. Statistical evaluation was performed by one-way ANOVA with Bonferroni post-hoc (*p≤0.05).
(TIF)

S1 Table Comparison of microarray results versus CN-10. The table shows expression changes for stable clones 4–18 and 3–44 and the transient pool shRNA3T versus CN-10. Upregulated genes (≥ 2) and downregulated genes (≤ -2) are listed separately.
(XLS)
