# Correction: The Oncogene PDRG1 Is an Interaction Target of Methionine Adenosyltransferases

**DOI:** 10.1371/journal.pone.0163761

**Published:** 2016-09-22

**Authors:** Claudia Pérez, Francisco J. Pérez-Zúñiga, Francisco Garrido, Edel Reytor, Francisco Portillo, María A. Pajares

The image for Fig 9 is incorrect. Please see the corrected Fig 9 here.

**Fig 9:**
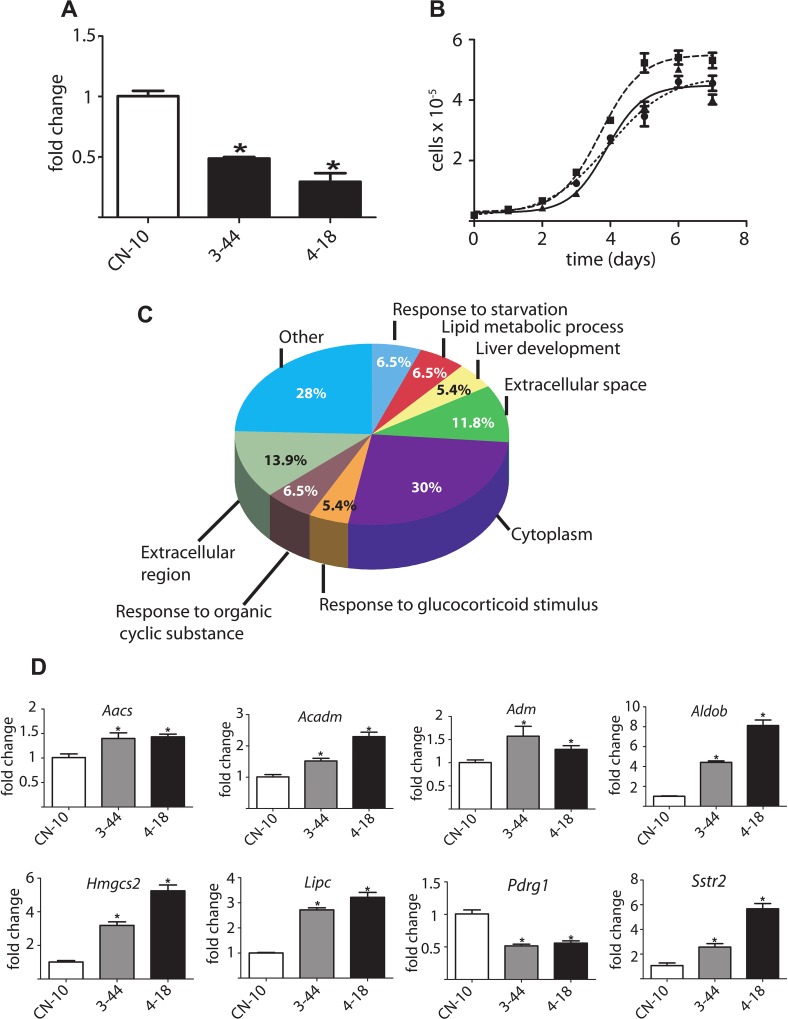
Differential expression analysis of *Pdrg1* silenced clones prepared in H35 cells. **(A**) Real-time RT-PCR analysis of *Pdrg1* expression using the *18s* gene as reference in the stable silenced clones (3–44 and 4–18) and the negative control clone (CN-10) prepared in H35 cells. The results shown are the mean ± SEM of four independent experiments carried out in triplicate. **(B)** Growth curves for H35 wild type cells (♦), the CN-10 (■) and 4–18 (●) clones; the figure shows the mean ± SEM of eight replicates of a representative independent experiment from the four carried out. **(C)** Pathway analysis of genes exhibiting expression changes ≥2-fold using Gene Ontology; only pathways with p<0.05 are indicated. **(D)** Real-time RT-PCR verification of expression changes (mean ± SEM; N = 4) in selected genes using the *Rn18s* gene as reference.
