# Supplementary figures and images for: The Oncogene PDRG1 Is an Interaction Target of Methionine Adenosyltransferases
Source: PLoS One. 2016 Aug 22;11(8):e0161672. doi: 10.1371/journal.pone.0161672 (PMC4993455; doi:10.1371/journal.pone.0161672)

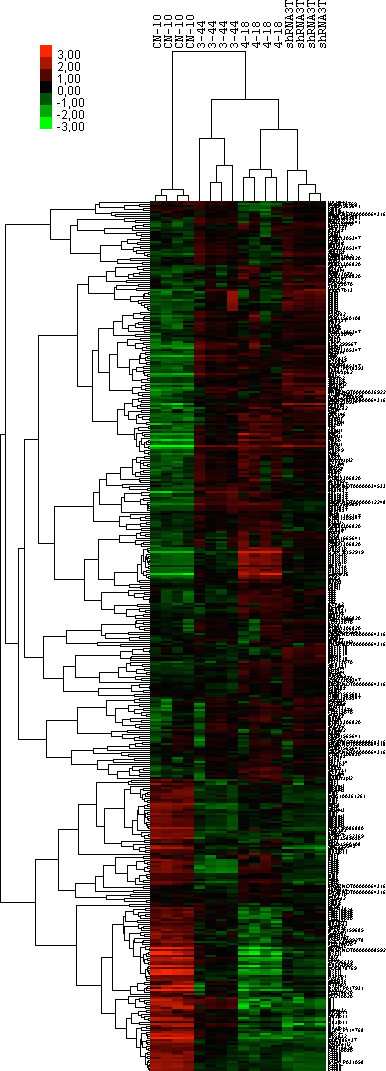

Supplement: S3 Fig — Microarray expression data were analyzed with FIESTA Viewer to identify genes exhibiting changes ≥2-fold with FDR<0.05. These data were used for clustering and preparation of heatmaps using Cluster and Java TreeView, respectively. The figure shows results (N = 4) of up- and down-regulated genes in stable clones CN-10, 3–44 and 4–18, as well as, in shRNA3T. (PNG) [file pone.0161672.s003.png]
